# Geographical distribution of typhoid risk factors in low and middle income countries

**DOI:** 10.1186/s12879-016-2074-1

**Published:** 2016-12-05

**Authors:** Jung-Seok Lee, Vijayalaxmi V. Mogasale, Vittal Mogasale, Kangsung Lee

**Affiliations:** International Vaccine Institute, SNU Research Park, 1 Gwanak-ro, Gwanack-gu, Seoul South Korea

**Keywords:** Typhoid, Risk factors, Composite index, Disease mapping

## Abstract

**Background:**

While the global burden of typhoid fever has been often brought up for attention, the detailed surveillance information has only been available for the limited number of countries. As more efficacious vaccines will be available in the near future, it is essential to understand the geographically diverse patterns of typhoid risk levels and to prioritize the right populations for vaccination to effectively control the disease.

**Methods:**

A composite index called the typhoid risk factor (TRF) index was created based on data with the Global Positioning System (GPS). Demographic and Health Surveys (DHS) and National Geographical Data Center (NGDC) satellite lights data were used for this analysis. A count model was adopted to validate the TRF index against the existing surveillance burden data. The TRF index was then re-estimated for 66 countries using the most recent data and mapped out for two geographical levels (sub-national boundary and grid-cell levels).

**Results:**

The TRF index which consists of drinking water sources, toilet facility types, and population density appeared to be statistically significant to explain variation in the disease burden data. The mapping analysis showed that typhoid risk levels vary not only by country but also by sub-national region. The grid-cell level analysis highlighted that the distribution of typhoid risk factors is uneven within the sub-national boundary level. Typhoid risk levels are geographically heterogeneous.

**Conclusions:**

Given the insufficient number of surveillance studies, the TRF index serves as a useful tool by capturing multiple risk factors of the disease into a single indicator. This will help decision makers identify high risk areas for typhoid as well as other waterborne diseases. Further, the study outcome can guide researchers to find relevant places for future surveillance studies.

**Electronic supplementary material:**

The online version of this article (doi:10.1186/s12879-016-2074-1) contains supplementary material, which is available to authorized users.

## Background

Typhoid fever remains a major public health concern in less developed countries [1–3]. The disease is transmitted through consumption of food or water contaminated with feces containing *Salmonella* Typhi. Typhoid is more common in impoverished areas with unsafe drinking water sources and poor sanitation. This can be exacerbated in areas where rapid population expansion is observed. More efficacious vaccines such as typhoid conjugate vaccines, are expected to be approved by World Health Organization (WHO) prequalification program in coming years. Considering many developing countries face limited resources and must contend with controlling typhoid transmission in endemic settings, it is critical to plan for the effective use of typhoid conjugate vaccines by prioritizing areas where people are at a greater risk for typhoid.

There are still large knowledge gaps about the disease burden of typhoid in many parts of developing countries. Community-based typhoid fever incidence studies are limited in number available from selected countries, and often confined to small areas such as urban slums. Typhoid disease is very common in South Asia because this is one of the regions where a large portion of the population lacks clean water and safe sanitation [4]. While much attention has been paid to urban or urban slum areas in South Asia [5–8], non-urban areas have received little attention with regards to typhoid occurrence, except for some selected studies [9–11]. In Latin America and the Caribbean, only two clinical trial data [12, 13] were found in a recent global typhoid burden study [3], and those studies were conducted over 20 years ago. This evidence gap is also apparent in Africa where there is high variability in incidence rates observed from published studies [14–18]. While low and medium incidence rates were reported in some countries [14–16], a recent population-based surveillance study in Kenya [18] revealed dramatically high typhoid incidence rates alerting critical knowledge gaps in the region where little attention has been paid. Although a multi-country burden study is currently underway in ten countries in Africa, a more evidence-based approach is needed to cover the relatively broader diversities in the region.

The previous global burden studies [1–3] have been useful to understand the extent of endemicity of typhoid. However it was inevitable for these models to rely on several assumptions due to the insufficient number of surveillance studies. Instead, this study proposes a new way of recognizing the global dynamics of exposure to typhoid infection by creating a composite index called the typhoid risk factor (TRF) index based on the fundamental risk factors of the disease. This study first attempts to identify typhoid risk factors which have strong relationships with typhoid incidence rates obtained from previous typhoid surveillance by taking into account the time and site location where each surveillance was conducted. Geocoding and spatial analysis techniques, which consider location and time information, have become an important tool in understanding various types of epidemiological trends over space. Geographical Information System and spatial statistics were previously used [4, 19, 20] and proved to be useful in identifying areas and populations at risk [4]. Once risk factors are determined, the study identifies populations exposed to different risk levels of typhoid infection including areas where no surveillance data is available.

## Methods

The overall study design consists of two parts: identification of typhoid risk factors and mapping out different risk levels using the most up-to-date data.

### Risk factor identification

**Fig. 1:**
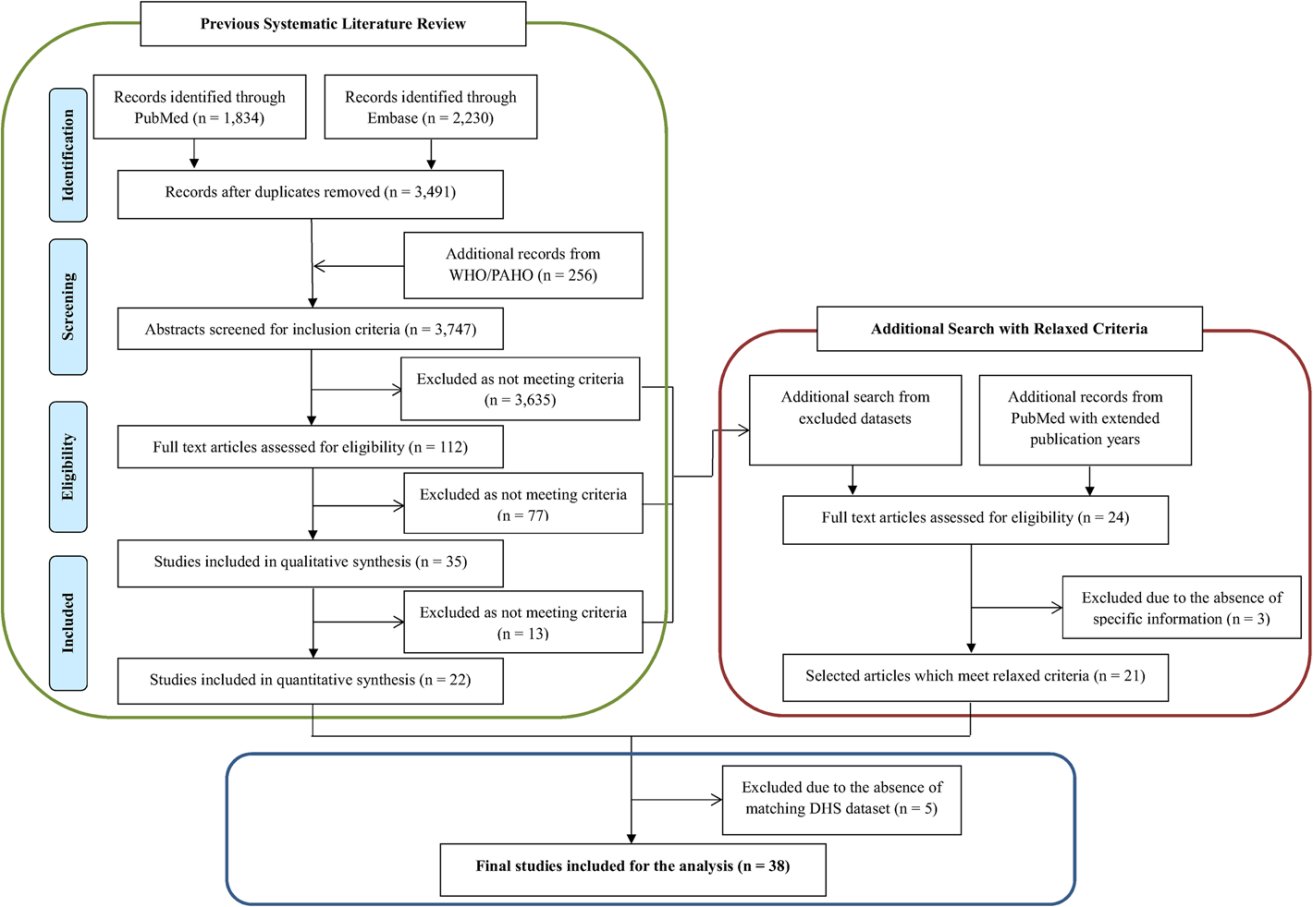
PRISMA

In order to identify risk factors for typhoid candidate risk factor variables were validated against typhoid disease burden data. A systematic literature review was previously conducted to establish a typhoid disease burden database [3]. To include more surveillance data than in the previous database, a second round of additional search was conducted with more relaxed criteria: extension of publication years from 1990–2013 to 1980–2013, inclusion of hospital-based studies, and community-based studies which did not meet the criteria in the previous search (e.g., blood culture, refusal rates). Thirty-eight studies were selected after considering the availability of matching data sources (Fig. 1), and these provided 84 disease burden data points by age group, as shown in Table 1.

**Table 1:** Disease burden data points by age group

Age group	Types of disease burden	
	Population based	Hospital based
Age group 1 (age < 2)	12	0
Age group 2 (2 ≤ age < 5)	17	2
Age group 3 (5 ≤ age < 15)	17	6
Age group 4 (age ≥ 15)	10	1
Age group 5 (overall)	15	4
Total	71	13

**Fig. 2:**
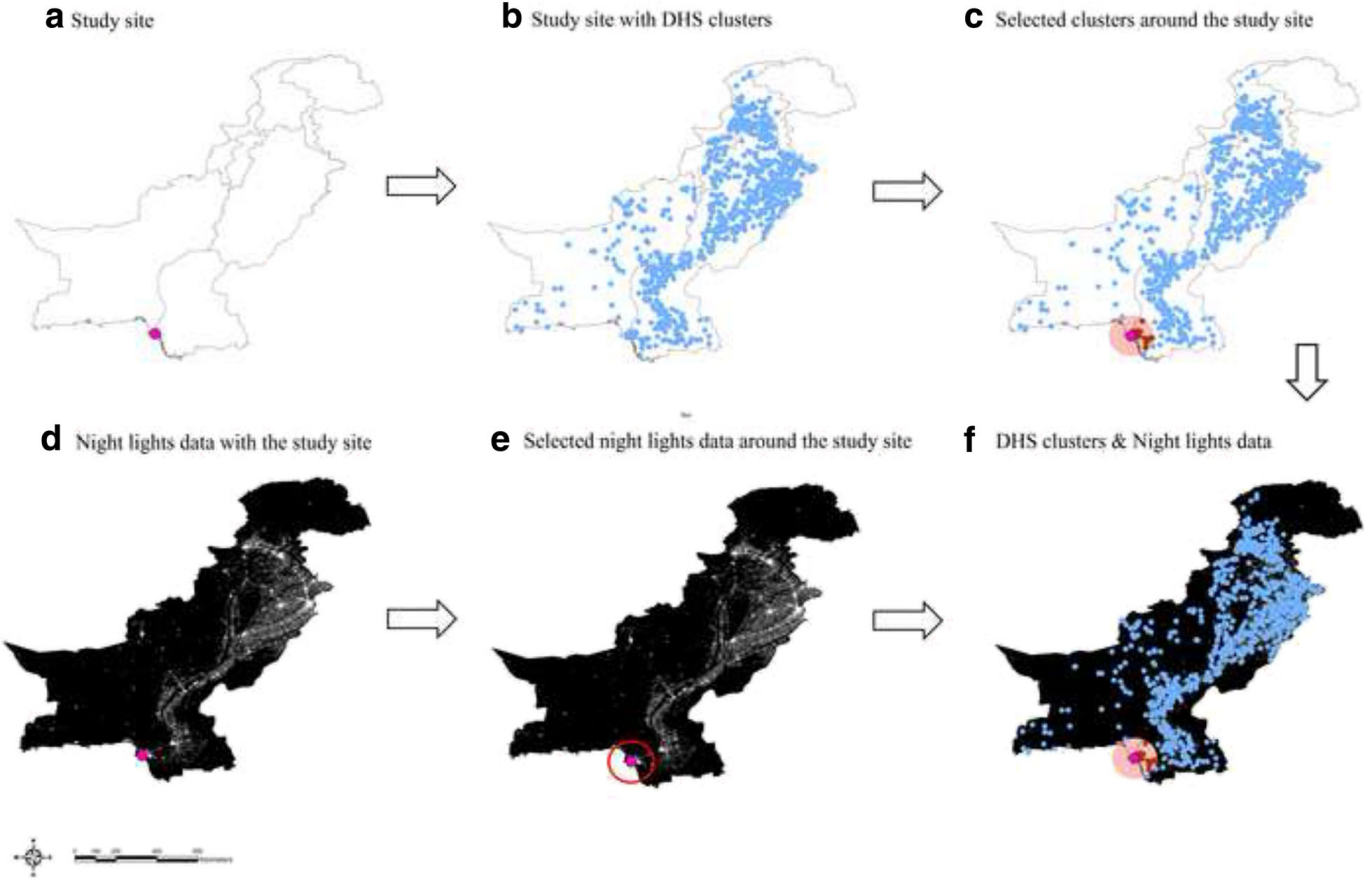
DHS and NGDC data inclusion process. **a** Study site. **b** Study site with DHS clusters. **c** Selected clusters around the study site. **d** Night lights data with the study site. **e** Selected night lights data around the study site. **f** DHS clusters & Night lights data

For candidate risk factors it was critical to find data sources available over time and across countries. Demographic and Health Surveys (DHS) and National Geographical Data Center (NGDC) satellite lights data were used for this analysis. Both DHS and NGDC provide not only longitudinal datasets, but also geo-coordinates of DHS clusters and NGDC lights data. Thus, each incidence rate estimated in a specific year and site was compared to candidate variables obtained from the same year of the surveillance (or close approximation) around the area where the surveillance was conducted. Figure 2 shows an example of Pakistan and demonstrates how both DHS and NGDC night lights data were paired with the disease burden data from the surveillance site. For example, clusters within 100 km from a site were included by using coordinates for the DHS dataset. For countries where there is no GPS information available, the next smallest geographical unit, which is the state-level in DHS, was used to select data in the state where the site was located. It is arbitrary whether the radius should be greater or less than 100 km. However, the distribution of DHS clusters is dispersed in some countries or states, whereas some clusters are densely gathered in other countries depending upon the size of countries or other logistical issues. Because this study includes multiple countries across continents, it is important to ensure that certain numbers of DHS clusters were included for all the surveillance sites. The 100 km radius ensures at least over 400 households for all the sites. This is a better way than pairing up with available country-level indicators ignoring the time and site location where surveillance was conducted.

It should be noted that although DHS carries out standardized surveys there are some differences across countries and survey periods in terms of questionnaire types and data availability. For this reason, variables which are most relevant to typhoid fever and exist consistently over time and across places were selected. As water and sanitation issues are the main risk factors for typhoid infection [19, 21–24], drinking water sources (hv201) and toilet facility types (hv205) were considered from the DHS dataset. Since the major categories of the variables are standard but individual codes are country-specific [25], the variables were sub-categorized as shown in Table 2. Each category was converted into a proportion out of total households who responded to each question. Prior to creating TRF indices and making formal comparisons, the sub-categories were first screened by simple scatter plots and correlations with the disease burden data by age group. The sub-categories which indicated unexpected signs or weak correlations (*ρ* < 0.4 *for hv*201, *ρ* < 0.3 *for hv*205) were excluded from further analysis.

**Table 2:** Sub-categorization for DHS variables

Category	HV201: source of drinking water	HV205: toilet facility types
1	Tubewell or borehole	Ventilated improved pit latrine
2	Protected well/spring, public covered well	Piped/septic sewer system, modern flush toilet
3	Unprotected well/spring, (public) open well	Pit latrine (covered: washable slab), composting
4	Well, spring, public well, hand pump	Pit latrine with non-washable slab/uncovered
5	Well in residence/yard/plot	Hanging latrine, drop/overhang
6	Piped water sources	Flush to others
7	Public/private tap water	Public/shared toilet, outside dwelling
8	Cart, buy from a car/vendor, tanker truck	Bucket, without cement sink, traditional toilet
9	Rain water	No facility, borehole, nature
10	Surface water	Others
11	Bottled (sachet) water	
12	Dam/river/lake/pond	
13	Others	

In addition population density was considered because highly crowded areas tend to have high rates of typhoid infection in developing countries [4, 6, 18]. In order to measure population density at the small geographical level over time, satellite lights data were obtained from NGDC. The satellite lights data are available from 1992 to 2012 in smaller geographical units: 30 arc second grid spacing (approximately 1 km^2^), and the same radius was used as DHS. The higher the lights were observed, the higher the population density was assumed. Prior to applying this assumption, correlations between lights data and census data were examined to ensure that lights data can be used as an appropriate proxy (overall ρ = 0.7).

The sub-categories filtered through the first screening (2 7, 9, 10, 11, 12 for hv201 and 1, 2, 3, 6, 7 for hv205) were used to create a composite index called the typhoid risk factor (TRF) index. To identify the most suitable index which explains the trend in typhoid incidence rates, five TRF indices were created based on different combinations of the sub-categories for the DHS variables and NGDC lights data. The variables in each set were first standardized individually by subtracting the mean and dividing by its standard deviation. The standardized values were then averaged across the variables. Some of the variables which go to the opposite direction were converted by subtracting from one, so all variables go towards the same underlying concept which is the typhoid risk level. For example, while a high proportion of the households who use river or lake as their water source would be at high risk, a high proportion of those who drink private tap water would be at low risk. The values were converted into a range from zero (low risk) to one (high risk) by using the max-min method and categorized into three percentiles (cTRF1: 0–25%, cTRF2: 25–75%, cTRF3: 75–100%). This method is more commonly used in the field of social science, and more details were extensively discussed elsewhere [26].

Incidence rates (/1000) can be considered as a non-negative integer value. Count models are suitable for our risk-factor validation because the count model estimates non-negative integer values and specifies the incidence rate with a mean that is dependent on exogenous variables [27, 28]. The Poisson or its variants (e.g., negative binomial) typically takes the exponential form for expected demand, and the Poisson probability density function can be written as

$$ \Pr \left({\mathrm{x}}_{\mathrm{i}}=\mathrm{n}\right)=\frac{{\mathrm{e}}^{-{\uplambda}_{\mathrm{i}}}{\uplambda}_{\mathrm{i}}^{\mathrm{n}}}{\mathrm{n}!},\kern0.75em \mathrm{n}=0,\ 1,\ 2\dots $$

where n is observed demand and λ_i_ is the mean, λ_i_ = exp(z_i_β). Overdispersion may occur when the variance is greater than the mean of the distribution [29, 30]. Additional file 1: Appendix 1 provides more details on how the overdispersion issue was managed. Care must be taken when dealing with counts of events observed in small geographical areas. While spatial correlation was less of a concern due to the data points obtained from dispersed geographical locations in this study, Moran I test was carried out to confirm that there is no significant spatial autocorrelation in the dataset [31, 32] (see Additional file 1: Appendix 1).

Because hospital-based incidence rates are likely to be higher than population-based incidence rates a dummy variable was created to treat them separately. This dummy variable was then multiplied by an age group categorical variable, and used as an interaction variable. Each of the five TRF indices was regressed against typhoid incidence rates separately, and the most statistically significant TRF index was chosen for the mapping analysis.

To understand the model fit the Akaike Information Criterion (AIC) and Bayesian Information Criterion (BIC) fit tests were used. Considering the relatively small sample size and generalizability of the model, a Hausman test (1978) on the equality of coefficients was carried out with 50% of randomly selected data from the main model [29]. The most statistically significant TRF index was chosen and used for further analyses.

### Mapping analyses

**Fig. 3:**
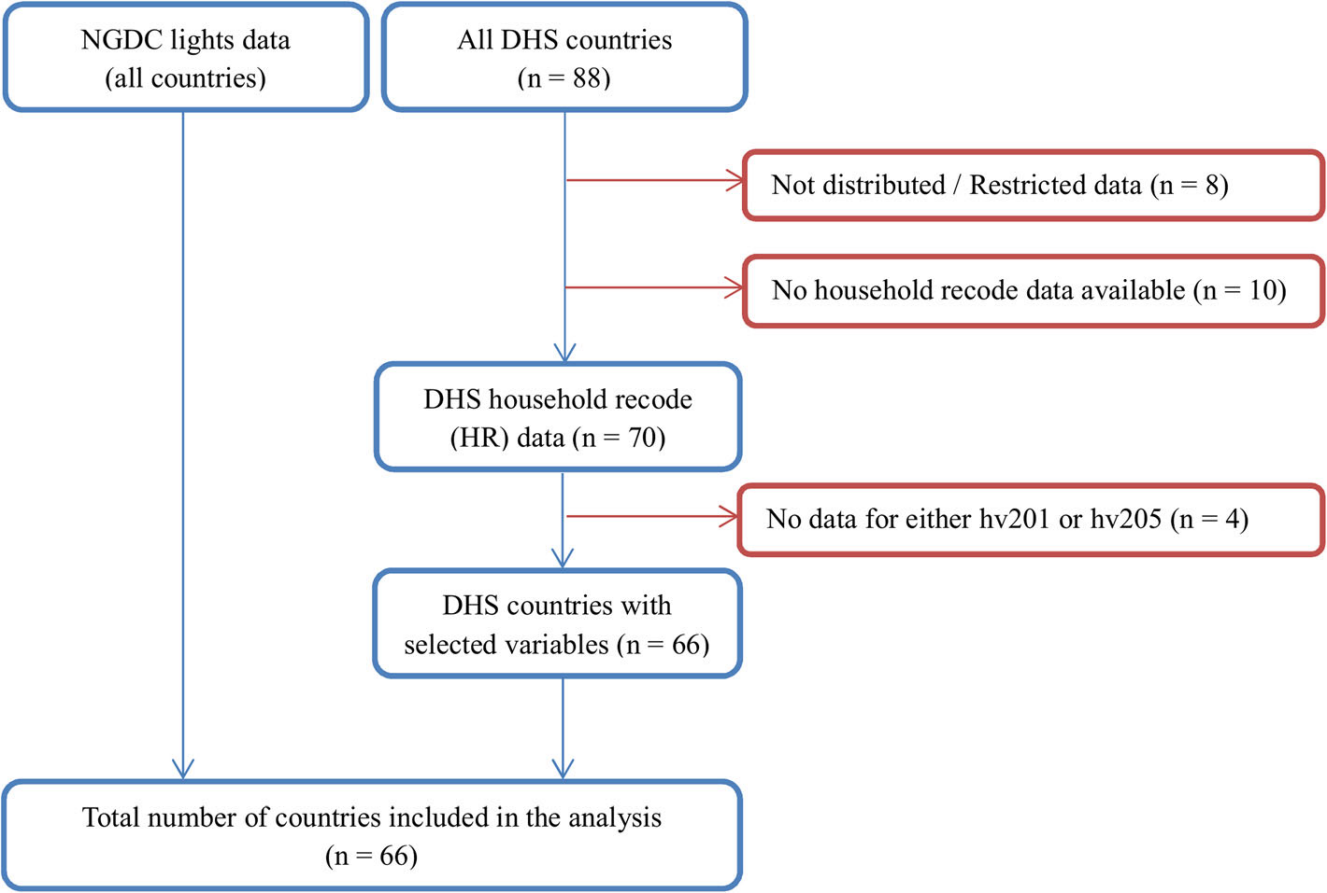
Country inclusion criteria

While a specific year of the datasets was considered to match the same period of each surveillance data for the validation model above the most recent datasets were obtained from the same data sources for the mapping analysis to show the current states of typhoid risk levels across countries. The final TRF index was re-estimated at two different levels: sub-national boundary level and grid-cell level. All countries where relevant information was available from both data sources were selected. Although NGDC provides lights data for all countries, DHS data were only available for the selected countries. As shown in Fig. 3, 66 out of 88 countries where drinking water sources and toilet facility types in the DHS household recode data exist were chosen for the mapping analysis.

The most recent years of DHS surveys vary country by country ranging from 1996 (Brazil) to 2013 (Nigeria). Because it would not be sensible to compare data in 1996 with the ones in 2013 the 66 DHS countries were divided into three groups (Table 3), and the same TRF index was estimated separately for each group.

**Table 3:** List of countries by DHS period

DHS year	Country
2010 – Present (*n* = 33)	Angola, Armenia, Bangladesh, Benin, Burkina Faso, Burundi, Cameroon, Colombia, Comoros, Congo Rep, Cote d’Ivoire, Ethiopia, Gabon, Guinea, Haiti, Honduras, Indonesia, Jordan, Kyrgyz Republic, Liberia, Madagascar, Malawi, Mozambique, Nepal, Niger, Nigeria, Rwanda^a^, Senegal^a^, Tajikistan, Tanzania, Timor-Leste, Uganda, Zimbabwe
2005–2009 (*n* = 22)	Albania, Azerbaijan, Congo Dem. Rep, Dominican Republic, Egypt, Ghana, Guyana, India, Kenya, Lesotho, Maldives, Moldova, Namibia, Pakistan^a^, Peru^a^, Philippines, Sao Tome and Principe, Sierra Leone, Swaziland, Ukraine, Vietnam, Zambia
Before 2005 (*n* = 11)	Brazil, Central African Republic, Chad, Guatemala, Kazakhstan, Morocco, Nicaragua, South Africa, Togo, Turkey, Yemen

^a^More recent years of surveys were available for these countries, but the most recent ones did not provide GPS information. Thus, the second recent datasets were chosen to have more sample size for the grid-cell analysis

**Fig. 4:**
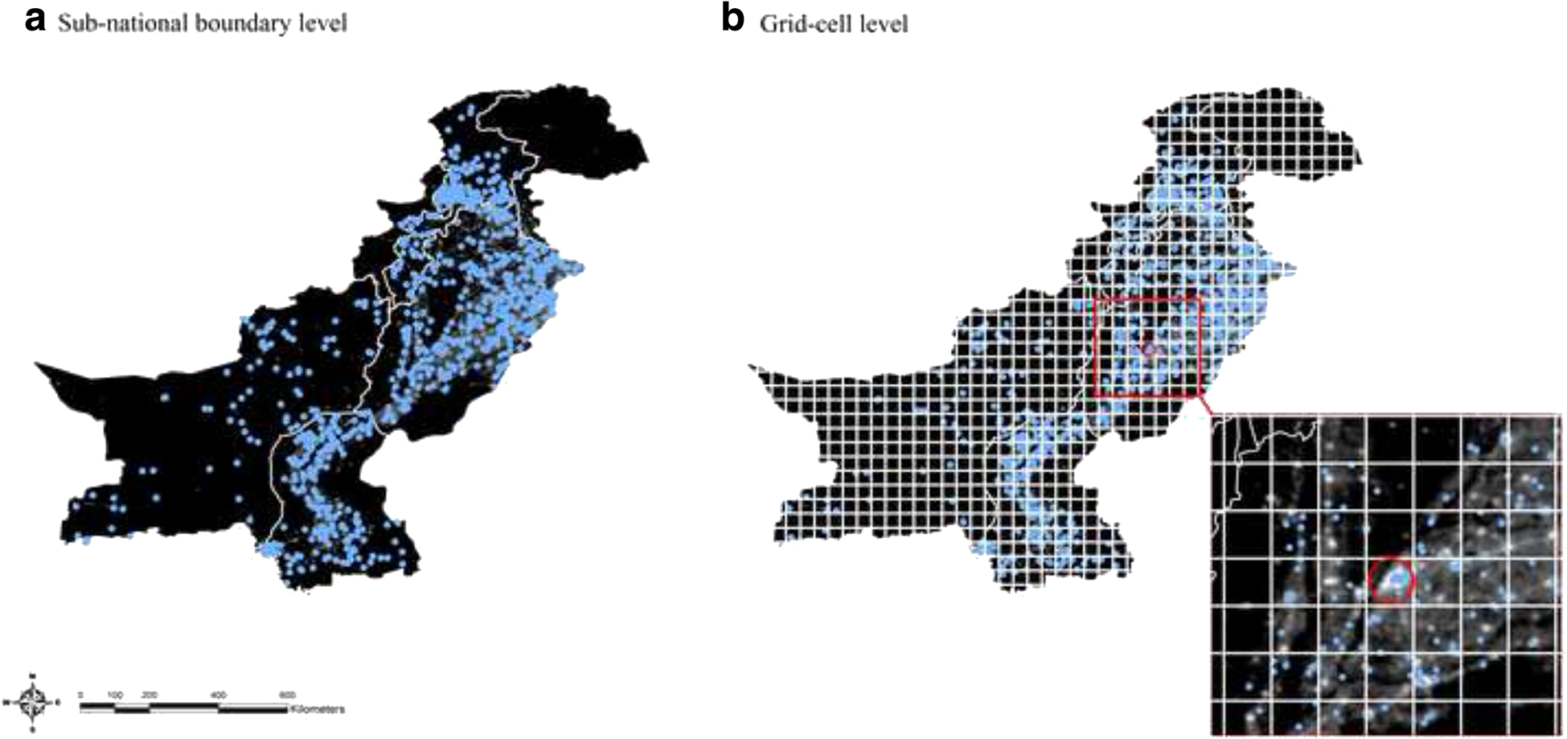
TRF index estimation by the sub-national boundary level and the grid-cell level in Pakistan. **a** Sub-national boundary level. **b** Grid-cell level

Figure 4 demonstrates how the TRF index was re-estimated by the sub-national boundary level and the grid-cell level. DHS provides sub-national boundaries which are usually the state or province level. This is the smallest geographical unit in which DHS sample weights were based. The proportions of sub-categories of the risk factor variables validated above were calculated by the DHS sub-national boundary level. The sample weights provided by DHS were applied so that the outcomes are representative at the population level. The mean value of lights data was also estimated by the sub-national boundary level for population density. The TRF index was then estimated by the same level. In addition, the grid-cell analysis was conducted in order to look at typhoid risk levels in the smaller geographical unit than the sub-national boundary level.. DHS provides geo-coordinates for the groups of households that participated in the survey, known as clusters for 46 of 66 countries. Considering the different sizes of countries and computational power, the size of grid-cells in each country was determined based on three categories: large (radius = 20.5 km), medium (radius = 10.5 km), and small (radius = 5 km). Centroids were created for all grid-cells. DHS clusters and NGDC lights data within a radius defined above from a centroid were included for each grid-cell. The risk factors were calculated in the same manner by the grid-cell level. It should be noted that unlike the sub-national boundary level, there were no sample weights applied in the grid-cell level analysis.

## Results

The validation model evaluated the predictors’ bearing on the incidence rate (/1000) for the three percentiles of the TRF index controlled by age group and types of incidence rates. The five TRF indices were regressed against typhoid incidence rates separately and compared in Table 4. A negative binomial model was preferred over a Poisson model after checking overdispersion in the data. Among the five types of TRF indices, the TRF index (type 5) with sub-category 4, 7, 11, 12 for hv201, 6 for hv205, and population density data appeared to be the most statistically significant index to explain variation in the disease burden data, after reversing the sub-categories of 7, 11 for hv201, and 6 for hv205. This model also outperformed the others in terms of AIC and BIC. Table 4 shows regression coefficients for the risk factor identification analysis. cTRF1 (0–25%) and age group 5 were reference groups for each categorical variable. The TRF index (type 5) was statistically significant with expected signs for all categories. The age group variable and interaction variables were also highly significant. The Hausman test confirmed that we fail to reject the null hypothesis (*χ* ^2^ = 4.98, *P* = 0.89) that the coefficients of the two sets of data are the same, which validates the generalizability of the model. The full specification of each regression is available (see Additional file 1: Appendix 2).

**Table 4:** Regression output for the TRF validation model

Independent variables	Coefficients (SD)				
	TRF type 1 *(hv201: 9, 10, 12)*	TRF type 2 *(hv205: 2, 7)*	TRF type 3 *(hv201: 2, 7, 11)*	TRF type 4 *(hv205: 1, 3)*	TRF type 5 *(hv201: 4, 7, 11, 12, hv205: 6, NGDC)*
cTRF2 (25–75%)	-0.592 (0.31)^a^	-0.124 (0.404)	0.278 (0.326)	-0.368 (0.308)	1.165 (0.317)^c^
cTRF3 (75–100%)	0.379 (0.349)	0.179 (0.44)	-1 (0.453)^b^	0.029 (0.353)	1.422 (0.37)^c^
Age group 1 (age < 2)	0.952 (0.445)^b^	0.822 (0.437)^a^	0.798 (0.446)^a^	0.808 (0.44)^a^	0.791 (0.449)^a^
Age group 2 (2 ≤ age < 5)	-4.379 (1.337)^c^	-5.084 (1.327)^c^	-5.486 (1.383)^c^	-5.06 (1.346)^c^	-5.378 (1.337)^c^
Age group 3 (5 ≤ age < 15)	-1.639 (0.66)^b^	-1.745 (0.662)^c^	-1.513 (0.659)^b^	-1.505 (0.678)^b^	-1.705 (0.664)^b^
Age group 4 (age ≥ 15)	-3.686 (1.513)^b^	-4.39 (1.504)^c^	-5.086 (1.558)^c^	-4.485 (1.529)^c^	-4.944 (1.522)^c^
Incidence rate type dummy (population based vs. hospital based)	-3.278 (0.601)^c^	-3.396 (0.64)^c^	-4.139 (0.658)^c^	-3.391 (0.6)^c^	-3.58 (0.599)^c^
Interaction variable1 (age group 1 X incidence rate type dummy)	omitted	omitted	omitted	omitted	omitted
Interaction variable2 (age group 2 X incidence rate type dummy)	5.702 (1.39)^c^	6.277 (1.388)^c^	6.64 (1.439)^c^	6.294 (1.408)^c^	6.53 (1.393)^c^
Interaction variable3 (age group 3 X incidence rate type dummy)	2.357 (0.779)^c^	2.223 (0.776)^c^	1.905 (0.783)^b^	2.056 (0.785)^c^	2.236 (0.781)^c^
Interaction variable4 (age group 4 X incidence rate type dummy)	3.255 (1.597)^b^	4.005 (1.582)^b^	4.608 (1.637)^c^	4.188 (1.618)^c^	4.509 (1.605)^c^
Constant	3.521 (0.529)^c^	4.391 (0.512)^c^	5.086 (0.655)^c^	4.485 (0.58)^c^	3.521 (0.529)^c^
Log likelihood	-225.434	-230.207	-225.987	-229.632	-222.464
AIC	472.8678	482.4148	473.9739	481.2634	466.929
BIC	-243.245	-233.698	-242.139	-234.849	-249.184

^a^Significance at the 10% level, ^b^at the 5% level, ^c^at the 1% level

**Fig. 5:**
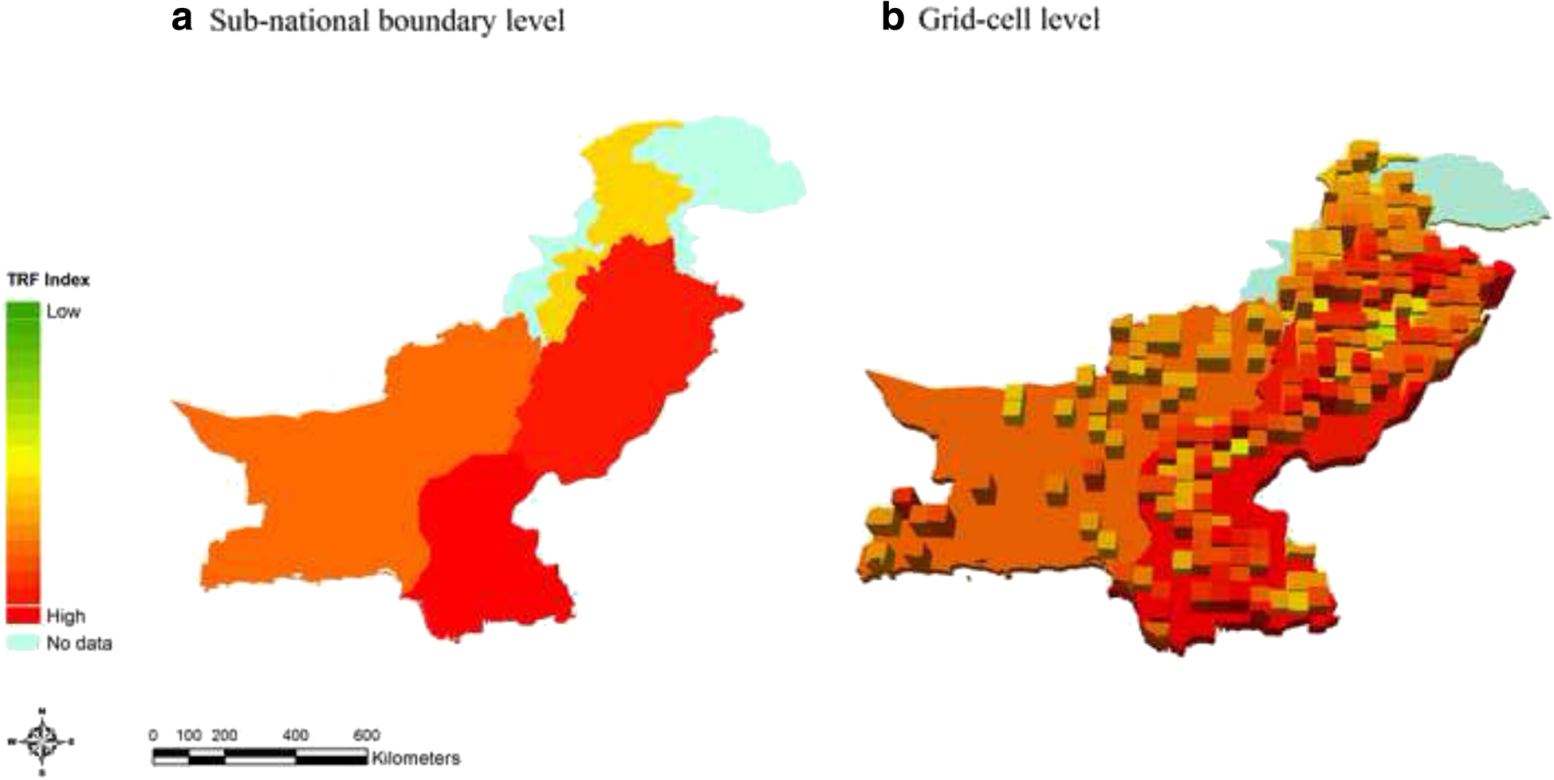
TRF index in Pakistan. **a** Sub-national boundary level. **b** Grid-cell level

The TRF index 5 was then selected as the final index and estimated for the mapping analysis to show the geographical distribution of typhoid risk levels. For interpretation-purposes the map of Pakistan was demonstrated in Fig. 5. The mapped color scale ranges from the low level of typhoid risk factors (dark green) to the high level of typhoid risk factors (dark red). The typhoid risk level estimated at the sub-national boundary level was very high in Sindh and Punjab as shown in Fig. 5(a). On the other hand, the North-Western frontier province was relatively moderate compared to other provinces in terms of the risk level. The grid-cell analysis was also conducted for Pakistan and demonstrated in Fig. 5(b), showing that typhoid risk levels vary not only by province but also within each province. In Sindh and Punjab, most of the households located in the grid-cells were highly exposed to typhoid risk factors, reflecting the overall risk level of the province. However, some areas in Punjab were safer than some places in the North-Western frontier province. This provides information that not all households are at high risk for typhoid in the province where the overall sub-national boundary level TRF index is high in the nation. Thus, the grid-cell analysis helps target high risk areas more precisely when resources for vaccination are limited.

**Fig. 6:**
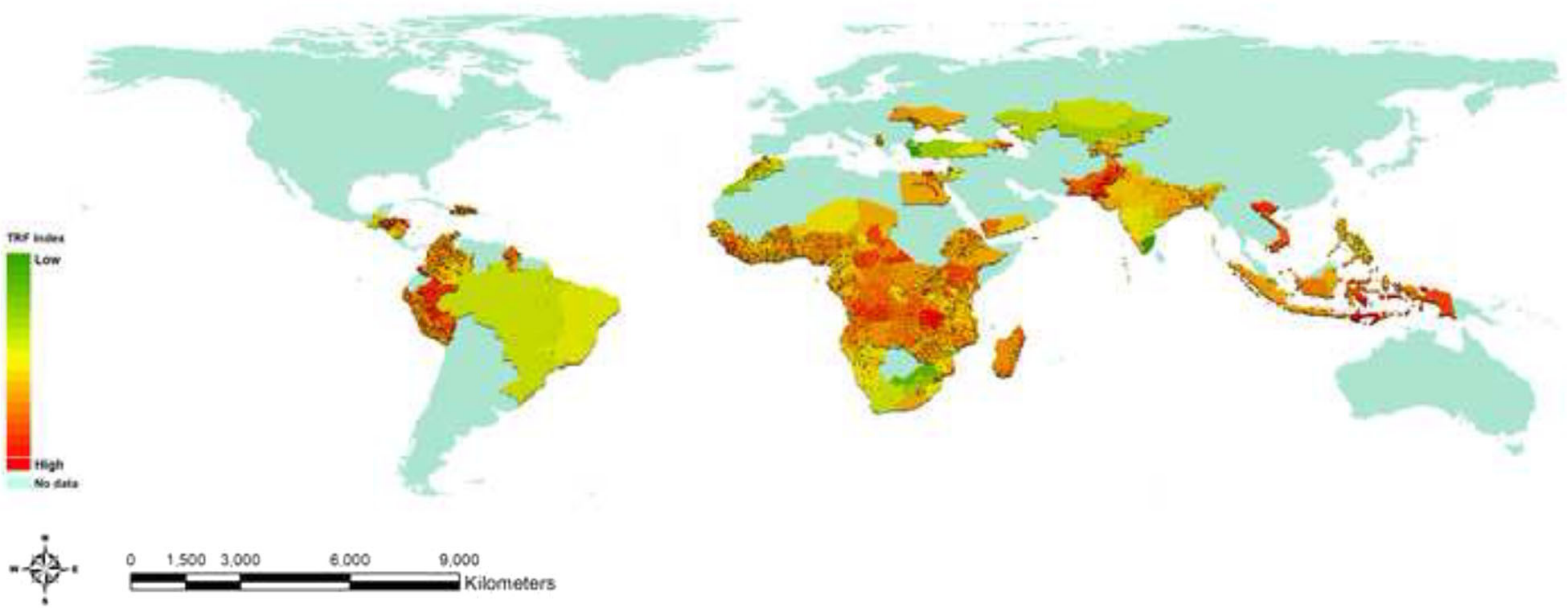
TRF index for the countries included in the study

Similarly the global distribution of typhoid risk levels as estimated by the TRF index 5 was shown in Fig. 6. 37 of the 66 countries were from the African region. Of these, the states at high risk for typhoid (TRF index > 0.9) include the Northern province in Sierra Leone, Luapula and Northern provinces in Zambia. In a total of 16 countries in Asia, East Nusa Tenggara in Indonesia, Punjab and Sindh in Pakistan were at high risk for typhoid. Among 13 countries from Latin America, Caribbean, and Europe, the states with the TRF index values above the same threshold were Puno and Ucayali in Peru, Gracias a Dios in Honduras, and Urban Tirana in Albania. It is worth noting that little attention has been paid to Latin America compared to other regions, partly due to the significant improvement in water and sanitation infrastructure over the past decade. However, some countries such as Honduras and Peru still appeared to be at high risk for typhoid. The grid-cell analysis was carried out for 31 countries in Africa, 7 in Asia, and 8 in Latin America and others. Additional file 1: Appendix 3 demonstrates the maps for the TRF index by regional group (see Additional file 1: Appendix 3). A full list of the TRF index by sub-national boundary is available (see Additional file 1: Appendix 4).

## Discussion

This study provides insight into the identification of typhoid risk factors by finding the relationships between surveillance data and socio-environmental circumstances. Some of the risk factors identified in this study were consistent with previous findings [33, 34]. In particular, Dewan et al. found that communities closer to rivers have elevated risk of typhoid infection compared to other locations in Bangladesh [33]. The TRF index constructed in this study summarized multiple risk factors of the disease as a single indicator which enables people to interpret easily. This approach is useful to maximize the effective use of typhoid conjugate vaccines to control typhoid in endemic settings where vaccination strategies should be carefully determined. Because many endemic countries do not have sufficient resources to fund mass vaccination programs, decision makers may need to identify relatively small, yet well-defined geographical locations to prioritize vaccinating populations at high risk areas [33].

The TRF index expressed through the mapping analysis identifies high risk areas and can be a useful tool for decision makers to prioritize target populations for vaccination. The TRF index is a relative measure for the countries included in this study. Thus the TRF index at the sub-national boundary level reflects the overall typhoid risk level of a state (or province) relative to other states. At the grid-cell level, different dynamics of risk level were further specified in the state. For example, the overall risk level of the Dhaka state in Bangladesh is relatively lower than those of other states. This does not necessarily mean that typhoid incidence is low in the entire area of Dhaka. As shown in the grid-cell level analysis, selected areas in Dhaka are still at high risk for typhoid (see Additional file 1: Appendix 5). In fact, Corner et al. [4] demonstrated that within Dhaka Metropolitan Area (DMA), 9.16% of population are at high risk, 44.01% are at moderate risk, and 46.83% are at low risk of typhoid showing that typhoid incidence varies in DMA.

The absence of surveillance data has been a consistent problem for various types of typhoid modeling studies at the global level and this study is not an exception. Despite the additional search with more relaxed criteria, the risk factor identification model would be more robust if more disease burden data points were available. Among the additional studies, some reported approximated population information, and this was manually adjusted for the surveillance periods, meaning the accuracy of the data would not be as robust as the data obtained from the literature review conducted beforehand. In order to assure the stability of the model, special care was taken in addition to model fit tests. Testing a model against validation data helps researchers to prevent from developing an overfitted model [29]. The Hausman test confirmed the generalizability of the model. To understand typhoid risk levels at the smaller geographical level than the sub-national boundary level, the grid-cell analysis was carried out for the countries where DHS GPS information is available. While the TRF index by the sub-national boundary level is representative at the population level, it should be noted that there were no sample weights available at the grid-cell level. Thus, typhoid risk level in the grid-cell analysis should be interpreted as the representativeness of the households in the clusters within a grid-cell, rather than the overall representativeness of a grid-cell. While the current study identified the six risk factors which consistently exist for all selected countries over time, others also found gender, health-seeking behavior, and seasonal variations as typhoid risk factors [33].

By using the TRF index the global disease burden of typhoid can be reformulated in a more sophisticated manner. In previous studies [1, 3], great efforts were made to measure the global typhoid burden. However, due to the limited amount of data sources, some broad assumptions were assigned to the point where the disease burden estimates from surveillance sites were regarded as the whole country-level, and some surveillance data were considered to be the same in neighboring countries where no such information was available. The recent burden study [3] updated the previous burden estimates by differentiating populations at high risk from non-high risk populations with an adjustment factor. However, this adjustment was also limited to applying a single odds ratio to all countries uniformly. In future studies, these limitations can be improved by adjusting surveillance data with the TRF index estimated in this study.

## Conclusions

While continuous efforts have been made over the past decades to estimate the different levels of typhoid disease burden, there are still large knowledge gaps which leave the typhoid burden in many parts of developing countries unknown. The TRF index and mapping analysis proposed in this study can facilitate the process of targeting appropriate populations in high risk areas for typhoid fever prevention activities such as vaccination. As typhoid conjugate vaccines will be available in the near future, our study findings can help decision makers in resource-constrained countries plan more effective vaccination strategies at the local level and can also ease potential supply limitations during the early stage of the new vaccine introduction. Given that many parts of developing countries still lacks population-based surveillance data, this study can guide decision makers in identifying areas where future surveillance studies should be conducted. Furthermore, because the study outcomes were generated based on public data sources which are periodically updated, these findings can assess the progress of the countries over time by observing changes in the index values as the information is updated.

## Additional file

Additional file 1: Appendix 1. Overdispersion. **Appendix 2.** Full specification of regression outputs. **Appendix 3.** TRF index (type 5) by sub-national boundary. **Appendix 4.** Final TRF index values by sub-national boundary. **Appendix 5.** TRF index in Bangladesh. (ZIP 1995 kb)

## Abbreviations

TRF: Typhoid risk factor
GPS: Global Positioning System
DHS: Demographic and Health Surveys
NGDC: National Geographical Data Center
AIC: Akaike Information Criterion
BIC: Bayesian Information Criterion
DMA: Dhaka Metropolitan Area
