# Supplementary material for: Geographical distribution of typhoid risk factors in low and middle income countries
Source: BMC Infect Dis. 2016 Dec 5;16:732. doi: 10.1186/s12879-016-2074-1 (PMC5139008; doi:10.1186/s12879-016-2074-1)
Supplement: Additional file 1: Appendix 1. — Overdispersion. Appendix 2. Full specification of regression outputs. Appendix 3. TRF index (type 5) by sub-national boundary. Appendix 4. Final TRF index values by sub-national boundary. Appendix 5. TRF index in Bangladesh. (ZIP 1995 kb) [file 12879_2016_2074_MOESM1_ESM.zip › Supplementary_v3R2.docx]

**Appendix 1. Overdispersion**

For the Poisson model, the mean is equal to the variance of the distribution. If the variance is greater than the mean, the model is mis-specified due to overdispersion ([29](#_ENREF_29), [30](#_ENREF_30)). Overdispersion may not affect the coefficient estimates significantly, but causes standard errors of the estimates to be underestimated. For this reason, the Z-score test and the boundary likelihood ratio test were performed to test for overdispersion ([29](#_ENREF_29)).

The Z-score tests the amount of overdispersion in a Poisson model to see whether it violates the basic assumptions ([29](#_ENREF_29)):

$$Z_{i}=\frac{\left( n_{i}-\mu_{i} \right)^{2}-n_{i}}{\mu_{i}\sqrt{2}}$$

where $n_{i}$ is observed values, $\mu_{i}$ is predicted values.

As shown in table A1-1, the Z-score test is 3.51 with a t-probability of 0.002. The null hypothesis that there is no overdispersion is rejected, and a negative binomial model is preferred to a Poisson model.

**A1-1. Z-score test**

| **Z** | **Coeff.** | **Std. Err.** | **T** | **P>\|t\|** | **95% Conf. Interval** | |
| --- | --- | --- | --- | --- | --- | --- |
|  | 3.51 | 1.07 | 3.27 | 0.002 | 1.36 | 5.64 |

The boundary likelihood ratio test is to evaluate if the negative binomial heterogeneity parameter ($\alpha$) is significantly greater than 0 ([29](#_ENREF_29)):

$$LR=-2\left( \mathcal{L}_{P}-\mathcal{L}_{\mathrm{NB}} \right)$$

where $\mathcal{L}_{P}$ is a Poisson log-likelihood estimate, and $\mathcal{L}_{\mathrm{NB}}$ is an negative binomial log-likelihood estimate. The p-value is defined as “one half the probability that a chi2 value (degree of freedom = 1) is greater than the obtained likelihood ratio statistic”. The null hypothesis that there is no overdispersion in the dataset is again rejected with the p-value (= 0.000).

Overdispersion may occur due to spatial autocorrelation when dealing with counts of events observed in small geographical areas. Moran’s I is a measure of spatial autocorrelation and defined as below:

$$I=\frac{\sum_{i=1}^{N} \sum_{j=1}^{N} w_{ij}\left( y_{i}-\bar{y} \right)\left( y_{j}-\bar{y} \right)}{\frac{1}{N}\sum_{i=1}^{N} \left( y_{i}-\bar{y} \right)^{2}\sum_{i=1}^{N} \sum_{j=1}^{N} w_{ij}}$$

where $y_{i}$ is the value in region $r_{i}$, $y_{j}$ is the value in $y_{j}$, $\bar{y}$ is the average value of Y, and $w_{ij}$ is the degree of proximity (weight) between regions $r_{i}$ and $r_{j}$ [[3](#_ENREF_3)]. Based on the results in table A1-2, we fail to reject the null hypothesis that there is zero spatial autocorrelation present in our dataset at the 0.05 level.

**A1-2. Moran's I**

| **Variables** | **I** | **E(I)** | **sd(I)** | **z** | **p-value^*^** |
| --- | --- | --- | --- | --- | --- |
| Incidence rates (/1,000) | 0.031 | -0.014 | 0.066 | 0.680 | 0.248 |
| TRF5 | -0.022 | -0.014 | 0.072 | -0.107 | 0.457 |

*1-tail test

**Appendix 2. Full specification of regression outputs**

**A2. Regression outputs**

(a) TRF index type 1

| **Independent variables** | **Coeff.** | **Std. Err.** | **z** | **P > \|z\|** | **95% CI** | |
| --- | --- | --- | --- | --- | --- | --- |
|  |  |  |  |  | **Lower** | **Upper** |
| cTRF2 | -0.592 | 0.310 | -1.91 | 0.056 | -1.200 | 0.016 |
| cTRF3 | 0.379 | 0.349 | 1.08 | 0.278 | -0.306 | 1.064 |
| Age group 1 (age < 2) | 0.952 | 0.445 | 2.14 | 0.033 | 0.079 | 1.825 |
| Age group 2 (2 ≤ age < 5 ) | -4.379 | 1.337 | -3.28 | 0.001 | -7.000 | -1.758 |
| Age group 3 (5 ≤ age < 15) | -1.640 | 0.660 | -2.48 | 0.013 | -2.933 | -0.346 |
| Age group 4 (age ≥ 15) | -3.686 | 1.513 | -2.44 | 0.015 | -6.651 | -0.721 |
| Incidence rate type dummy (population based vs. hospital based) | -3.278 | 0.601 | -5.45 | 0.000 | -4.456 | -2.100 |
| Interaction variable1 (age group 1 X incidence rate type dummy) | omitted |  |  |  |  |  |
| Interaction variable2 (age group 2 X incidence rate type dummy) | 5.702 | 1.391 | 4.1 | 0.000 | 2.976 | 8.427 |
| Interaction variable3 (age group 3 X incidence rate type dummy) | 2.357 | 0.779 | 3.03 | 0.002 | 0.831 | 3.883 |
| Interaction variable4 (age group 4 X incidence rate type dummy) | 3.255 | 1.597 | 2.04 | 0.041 | 0.126 | 6.385 |
| Constant | 4.278 | 0.585 | 7.31 | 0.000 | 3.131 | 5.425 |
| N | 84 |  |  |  |  |  |
| Deviance | 80.205006 |  |  |  |  |  |
| Pearson | 71.00797 |  |  |  |  |  |
| Log likelihood | -225.434 |  |  |  |  |  |
| AIC | 472.868 |  |  |  |  |  |
| BIC | -243.245 |  |  |  |  |  |

(b) TRF index type 2

| **Independent variables** | **Coeff.** | **Std. Err.** | **z** | **P > \|z\|** | **95% CI** | |
| --- | --- | --- | --- | --- | --- | --- |
|  |  |  |  |  | **Lower** | **Upper** |
| cTRF2 | -0.124 | 0.405 | -0.31 | 0.759 | -0.917 | 0.669 |
| cTRF3 | 0.179 | 0.440 | 0.41 | 0.684 | -0.684 | 1.042 |
| Age group 1 (age < 2) | 0.822 | 0.437 | 1.88 | 0.060 | -0.034 | 1.679 |
| Age group 2 (2 ≤ age < 5 ) | 5.084 | 1.327 | -3.83 | 0.000 | -7.685 | -2.482 |
| Age group 3 (5 ≤ age < 15) | 1.745 | 0.662 | -2.63 | 0.008 | -3.043 | -0.447 |
| Age group 4 (age ≥ 15) | -4.391 | 1.504 | -2.92 | 0.004 | -7.338 | -1.443 |
| Incidence rate type dummy (population based vs. hospital based) | -3.396 | 0.640 | -5.31 | 0.000 | -4.650 | -2.142 |
| Interaction variable1 (age group 1 X incidence rate type dummy) | omitted |  |  |  |  |  |
| Interaction variable2 (age group 2 X incidence rate type dummy) | 6.277 | 1.388 | 4.52 | 0.000 | 3.557 | 8.996 |
| Interaction variable3 (age group 3 X incidence rate type dummy) | 2.223 | 0.776 | 2.87 | 0.004 | 0.703 | 3.743 |
| Interaction variable4 (age group 4 X incidence rate type dummy) | 4.005 | 1.582 | 2.53 | 0.011 | 0.905 | 7.106 |
| Constant | 4.391 | 0.512 | 8.58 | 0.000 | 3.387 | 5.394 |
| N | 84 |  |  |  |  |  |
| Deviance | 89.752 |  |  |  |  |  |
| Pearson | 76.595 |  |  |  |  |  |
| Log likelihood | -230.207 |  |  |  |  |  |
| AIC | 482.415 |  |  |  |  |  |
| BIC | -233.698 |  |  |  |  |  |

(c) TRF index type 3

| **Independent variables** | **Coeff.** | **Std. Err.** | **z** | **P > \|z\|** | **95% CI** | |
| --- | --- | --- | --- | --- | --- | --- |
|  |  |  |  |  | **Lower** | **Upper** |
| cTRF2 | 0.278 | 0.326 | 0.85 | 0.393 | -0.360 | 0.916 |
| cTRF3 | -1.000 | 0.453 | -2.21 | 0.027 | -1.888 | -0.112 |
| Age group 1 (age < 2) | 0.798 | 0.446 | 1.79 | 0.074 | -0.076 | 1.673 |
| Age group 2 (2 ≤ age < 5 ) | -5.486 | 1.383 | -3.97 | 0.000 | -8.197 | -2.776 |
| Age group 3 (5 ≤ age < 15) | -1.513 | 0.659 | -2.3 | 0.022 | -2.805 | -0.222 |
| Age group 4 (age ≥ 15) | -5.086 | 1.558 | -3.26 | 0.001 | -8.140 | -2.031 |
| Incidence rate type dummy (population based vs. hospital based) | -4.139 | 0.658 | -6.29 | 0.000 | -5.429 | -2.850 |
| Interaction variable1 (age group 1 X incidence rate type dummy) | omitted |  |  |  |  |  |
| Interaction variable2 (age group 2 X incidence rate type dummy) | 6.640 | 1.439 | 4.61 | 0.000 | 3.819 | 9.460 |
| Interaction variable3 (age group 3 X incidence rate type dummy) | 1.905 | 0.783 | 2.43 | 0.015 | 0.371 | 3.440 |
| Interaction variable4 (age group 4 X incidence rate type dummy) | 4.608 | 1.637 | 2.82 | 0.005 | 1.400 | 7.816 |
| Constant | 5.086 | 0.655 | 7.77 | 0.000 | 3.803 | 6.368 |
| N | 84 |  |  |  |  |  |
| Deviance | 81.311 |  |  |  |  |  |
| Pearson | 68.851 |  |  |  |  |  |
| Log likelihood | -225.987 |  |  |  |  |  |
| AIC | 473.9739 |  |  |  |  |  |
| BIC | -242.139 |  |  |  |  |  |

(d) TRF index type 4

| **Independent variables** | **Coeff.** | **Std. Err.** | **z** | **P > \|z\|** | **95% CI** | |
| --- | --- | --- | --- | --- | --- | --- |
|  |  |  |  |  | **Lower** | **Upper** |
| cTRF2 | -0.368 | 0.308 | -1.19 | 0.233 | -0.972 | 0.237 |
| cTRF3 | 0.029 | 0.353 | 0.08 | 0.935 | -0.663 | 0.721 |
| Age group 1 (age < 2) | 0.808 | 0.440 | 1.83 | 0.067 | -0.056 | 1.671 |
| Age group 2 (2 ≤ age < 5 ) | -5.060 | 1.346 | -3.76 | 0.000 | -7.698 | -2.423 |
| Age group 3 (5 ≤ age < 15) | -1.505 | 0.678 | -2.22 | 0.027 | -2.834 | -0.175 |
| Age group 4 (age ≥ 15) | -4.485 | 1.529 | -2.93 | 0.003 | -7.481 | -1.489 |
| Incidence rate type dummy (population based vs. hospital based) | -3.391 | 0.600 | -5.65 | 0.000 | -4.567 | -2.215 |
| Interaction variable1 (age group 1 X incidence rate type dummy) | omitted |  |  |  |  |  |
| Interaction variable2 (age group 2 X incidence rate type dummy) | 6.294 | 1.408 | 4.47 | 0.000 | 3.535 | 9.053 |
| Interaction variable3 (age group 3 X incidence rate type dummy) | 2.056 | 0.785 | 2.62 | 0.009 | 0.517 | 3.596 |
| Interaction variable4 (age group 4 X incidence rate type dummy) | 4.188 | 1.618 | 2.59 | 0.010 | 1.017 | 7.359 |
| Constant | 4.485 | 0.580 | 7.73 | 0.000 | 3.347 | 5.622 |
| N | 84 |  |  |  |  |  |
| Deviance | 88.601 |  |  |  |  |  |
| Pearson | 74.207 |  |  |  |  |  |
| Log likelihood | -229.632 |  |  |  |  |  |
| AIC | 481.2634 |  |  |  |  |  |
| BIC | -234.849 |  |  |  |  |  |

(e) TRF index type 5

| **Independent variables** | **Coeff.** | **Std. Err.** | **z** | **P > \|z\|** | **95% CI** | |
| --- | --- | --- | --- | --- | --- | --- |
|  |  |  |  |  | **Lower** | **Upper** |
| cTRF2 | 1.165 | 0.317 | 3.67 | 0.000 | 0.543 | 1.787 |
| cTRF3 | 1.422 | 0.370 | 3.85 | 0.000 | 0.698 | 2.147 |
| Age group 1 (age < 2) | 0.791 | 0.449 | 1.76 | 0.078 | -0.088 | 1.671 |
| Age group 2 (2 ≤ age < 5 ) | -5.379 | 1.337 | -4.02 | 0.000 | -7.998 | -2.759 |
| Age group 3 (5 ≤ age < 15) | -1.705 | 0.664 | -2.57 | 0.010 | -3.006 | -0.403 |
| Age group 4 (age ≥ 15) | -4.944 | 1.522 | -3.25 | 0.001 | -7.927 | -1.960 |
| Incidence rate type dummy (population based vs. hospital based) | -3.581 | 0.599 | -5.97 | 0.000 | -4.756 | -2.406 |
| Interaction variable1 (age group 1 X incidence rate type dummy) | omitted |  |  |  |  |  |
| Interaction variable2 (age group 2 X incidence rate type dummy) | 6.529 | 1.393 | 4.69 | 0.000 | 3.799 | 9.259 |
| Interaction variable3 (age group 3 X incidence rate type dummy) | 2.236 | 0.782 | 2.86 | 0.004 | 0.704 | 3.768 |
| Interaction variable4 (age group 4 X incidence rate type dummy) | 4.509 | 1.606 | 2.81 | 0.005 | 1.362 | 7.656 |
| Constant | 3.521 | 0.529 | 6.66 | 0.000 | 2.484 | 4.558 |
| N | 84 |  |  |  |  |  |
| Deviance | 74.266 |  |  |  |  |  |
| Pearson | 51.037 |  |  |  |  |  |
| Log likelihood | -222.464 |  |  |  |  |  |
| AIC | 466.929 |  |  |  |  |  |
| BIC | -249.184 |  |  |  |  |  |

**Appendix 3. TRF index (type 5) by sub-national boundary**

**A3-1. TRF index in Africa**

**A3-2. TRF index in Central & South Asia**

**A3-3. TRF index in South-East Asia**

**A3-4. TRF index in Latin America**

**A3-5. TRF index in others**

**Appendix 4. Final TRF index values by sub-national boundary**

**A4. Final TRF index by sub-national boundary**

(a) Group 1 (2010 – present)

| **Continent** | **Region** | **Country** | **Sub-national boundary (by DHS)** | **TRF index** |
| --- | --- | --- | --- | --- |
| Africa | Central Africa | Angola | Hyperendemic | 0.87 |
| Africa | Central Africa | Angola | Luanda | 0.63 |
| Africa | Central Africa | Angola | Mesoendemic Stable | 0.74 |
| Africa | Central Africa | Angola | Mesoendemic Unstable | 0.69 |
| Africa | Central Africa | Cameroon | Adamawa | 0.57 |
| Africa | Central Africa | Cameroon | Centre | 0.57 |
| Africa | Central Africa | Cameroon | Douala | 0.52 |
| Africa | Central Africa | Cameroon | East | 0.58 |
| Africa | Central Africa | Cameroon | Far North | 0.63 |
| Africa | Central Africa | Cameroon | Littoral | 0.51 |
| Africa | Central Africa | Cameroon | North | 0.60 |
| Africa | Central Africa | Cameroon | Northwest | 0.57 |
| Africa | Central Africa | Cameroon | South | 0.56 |
| Africa | Central Africa | Cameroon | Southwest | 0.52 |
| Africa | Central Africa | Cameroon | West | 0.62 |
| Africa | Central Africa | Cameroon | Yaounde | 0.58 |
| Africa | Central Africa | Congo, Rep | Bouenza | 0.59 |
| Africa | Central Africa | Congo, Rep | Brazzaville | 0.70 |
| Africa | Central Africa | Congo, Rep | Cuvette | 0.70 |
| Africa | Central Africa | Congo, Rep | Cuvette-Ouest | 0.81 |
| Africa | Central Africa | Congo, Rep | Kouilou | 0.69 |
| Africa | Central Africa | Congo, Rep | Lekoumou | 0.59 |
| Africa | Central Africa | Congo, Rep | Likouala | 0.74 |
| Africa | Central Africa | Congo, Rep | Niari | 0.62 |
| Africa | Central Africa | Congo, Rep | Plateaux | 0.75 |
| Africa | Central Africa | Congo, Rep | Pointe-Noire | 0.70 |
| Africa | Central Africa | Congo, Rep | Pool | 0.69 |
| Africa | Central Africa | Congo, Rep | Sangha | 0.61 |
| Africa | Central Africa | Gabon | Estuaire | 0.57 |
| Africa | Central Africa | Gabon | Haut Ogooue | 0.55 |
| Africa | Central Africa | Gabon | Liberville/Port Gentil | 0.75 |
| Africa | Central Africa | Gabon | Moyen Ogooue | 0.60 |
| Africa | Central Africa | Gabon | Ngounie | 0.56 |
| Africa | Central Africa | Gabon | Nyanga | 0.59 |
| Africa | Central Africa | Gabon | Ogooue Ivindo | 0.63 |
| Africa | Central Africa | Gabon | Ogooue Lolo | 0.64 |
| Africa | Central Africa | Gabon | Ogooue Maritime | 0.59 |
| Africa | Central Africa | Gabon | Woleu Ntem | 0.53 |
| Africa | East Africa | Burundi | Bujumbura Mairie | 0.58 |
| Africa | East Africa | Burundi | Central East | 0.57 |
| Africa | East Africa | Burundi | North | 0.59 |
| Africa | East Africa | Burundi | South | 0.51 |
| Africa | East Africa | Burundi | West | 0.51 |
| Africa | East Africa | Comoros | Anjouan | 0.53 |
| Africa | East Africa | Comoros | Grande Comore | 0.54 |
| Africa | East Africa | Comoros | Moheli | 0.48 |
| Africa | East Africa | Ethiopia | Addis Ababa | 0.64 |
| Africa | East Africa | Ethiopia | Affar | 0.74 |
| Africa | East Africa | Ethiopia | Amhara | 0.62 |
| Africa | East Africa | Ethiopia | Benishangul-Gumuz | 0.65 |
| Africa | East Africa | Ethiopia | Dire Dawa | 0.48 |
| Africa | East Africa | Ethiopia | Gambela | 0.58 |
| Africa | East Africa | Ethiopia | Harari | 0.52 |
| Africa | East Africa | Ethiopia | Oromiya | 0.60 |
| Africa | East Africa | Ethiopia | SNNP | 0.63 |
| Africa | East Africa | Ethiopia | Somali | 0.62 |
| Africa | East Africa | Ethiopia | Tigray | 0.60 |
| Africa | East Africa | Madagascar | Equatorial | 0.75 |
| Africa | East Africa | Madagascar | Highlands | 0.59 |
| Africa | East Africa | Madagascar | Sub-Desert | 0.74 |
| Africa | East Africa | Madagascar | Tropical | 0.67 |
| Africa | East Africa | Malawi | Central | 0.58 |
| Africa | East Africa | Malawi | Northern | 0.62 |
| Africa | East Africa | Malawi | Southern | 0.56 |
| Africa | East Africa | Mozambique | Cabo Delgado | 0.59 |
| Africa | East Africa | Mozambique | Gaza | 0.52 |
| Africa | East Africa | Mozambique | Inhambane | 0.59 |
| Africa | East Africa | Mozambique | Manica | 0.50 |
| Africa | East Africa | Mozambique | Maputo | 0.37 |
| Africa | East Africa | Mozambique | Maputo City | 0.00 |
| Africa | East Africa | Mozambique | Nampula | 0.58 |
| Africa | East Africa | Mozambique | Niassa | 0.65 |
| Africa | East Africa | Mozambique | Sofala | 0.44 |
| Africa | East Africa | Mozambique | Tete | 0.67 |
| Africa | East Africa | Mozambique | Zambezia | 0.72 |
| Africa | East Africa | Rwanda | City of Kigali | 0.49 |
| Africa | East Africa | Rwanda | East | 0.60 |
| Africa | East Africa | Rwanda | North | 0.55 |
| Africa | East Africa | Rwanda | South | 0.59 |
| Africa | East Africa | Rwanda | West | 0.53 |
| Africa | East Africa | Tanzania | Arusha | 0.49 |
| Africa | East Africa | Tanzania | Dar es Salaam | 0.45 |
| Africa | East Africa | Tanzania | Dodoma | 0.53 |
| Africa | East Africa | Tanzania | Geita | 0.61 |
| Africa | East Africa | Tanzania | Iringa | 0.73 |
| Africa | East Africa | Tanzania | Kagera | 0.73 |
| Africa | East Africa | Tanzania | Kaskazini Pemba | 0.49 |
| Africa | East Africa | Tanzania | Kaskazini Unguja | 0.43 |
| Africa | East Africa | Tanzania | Katavi | 0.68 |
| Africa | East Africa | Tanzania | Kigoma | 0.54 |
| Africa | East Africa | Tanzania | Kilimanjaro | 0.43 |
| Africa | East Africa | Tanzania | Kusini Pemba | 0.46 |
| Africa | East Africa | Tanzania | Kusini Unguja | 0.46 |
| Africa | East Africa | Tanzania | Lindi | 0.64 |
| Africa | East Africa | Tanzania | Manyara | 0.69 |
| Africa | East Africa | Tanzania | Mara | 0.65 |
| Africa | East Africa | Tanzania | Mbeya | 0.56 |
| Africa | East Africa | Tanzania | Mjini Magharibi | 0.35 |
| Africa | East Africa | Tanzania | Morogoro | 0.57 |
| Africa | East Africa | Tanzania | Mtwara | 0.54 |
| Africa | East Africa | Tanzania | Mwanza | 0.52 |
| Africa | East Africa | Tanzania | Njombe | 0.61 |
| Africa | East Africa | Tanzania | Pwani | 0.60 |
| Africa | East Africa | Tanzania | Rukwa | 0.67 |
| Africa | East Africa | Tanzania | Ruvuma | 0.53 |
| Africa | East Africa | Tanzania | Shinyanga | 0.70 |
| Africa | East Africa | Tanzania | Simiyu | 0.71 |
| Africa | East Africa | Tanzania | Singida | 0.58 |
| Africa | East Africa | Tanzania | Tabora | 0.59 |
| Africa | East Africa | Tanzania | Tanga | 0.64 |
| Africa | East Africa | Uganda | Central 1 | 0.66 |
| Africa | East Africa | Uganda | Central 2 | 0.40 |
| Africa | East Africa | Uganda | East Central | 0.61 |
| Africa | East Africa | Uganda | Eastern | 0.60 |
| Africa | East Africa | Uganda | Kampala | 0.81 |
| Africa | East Africa | Uganda | Karamoja | 0.63 |
| Africa | East Africa | Uganda | North | 0.60 |
| Africa | East Africa | Uganda | Southwest | 0.63 |
| Africa | East Africa | Uganda | West Nile | 0.59 |
| Africa | East Africa | Uganda | Western | 0.65 |
| Africa | East Africa | Zimbabwe | Bulawayo | 0.68 |
| Africa | East Africa | Zimbabwe | Harare | 0.68 |
| Africa | East Africa | Zimbabwe | Manicaland | 0.60 |
| Africa | East Africa | Zimbabwe | Mashonaland Central | 0.63 |
| Africa | East Africa | Zimbabwe | Mashonaland East | 0.60 |
| Africa | East Africa | Zimbabwe | Mashonaland West | 0.61 |
| Africa | East Africa | Zimbabwe | Masvingo | 0.63 |
| Africa | East Africa | Zimbabwe | Matabeleland North | 0.62 |
| Africa | East Africa | Zimbabwe | Matabeleland South | 0.67 |
| Africa | East Africa | Zimbabwe | Midlands | 0.62 |
| Africa | West Africa | Benin | Alibori | 0.57 |
| Africa | West Africa | Benin | Atacora | 0.59 |
| Africa | West Africa | Benin | Atlantique | 0.51 |
| Africa | West Africa | Benin | Borgou | 0.52 |
| Africa | West Africa | Benin | Collines | 0.51 |
| Africa | West Africa | Benin | Couffo | 0.55 |
| Africa | West Africa | Benin | Donga | 0.55 |
| Africa | West Africa | Benin | Littoral | 0.76 |
| Africa | West Africa | Benin | Mono | 0.49 |
| Africa | West Africa | Benin | Oueme | 0.57 |
| Africa | West Africa | Benin | Plateau | 0.56 |
| Africa | West Africa | Benin | Zou | 0.53 |
| Africa | West Africa | Burkina Faso | Boucle du Mouhoun | 0.58 |
| Africa | West Africa | Burkina Faso | Cascades | 0.51 |
| Africa | West Africa | Burkina Faso | Centre | 0.50 |
| Africa | West Africa | Burkina Faso | Centre-Est | 0.59 |
| Africa | West Africa | Burkina Faso | Centre-Nord | 0.60 |
| Africa | West Africa | Burkina Faso | Centre-Ouest | 0.59 |
| Africa | West Africa | Burkina Faso | Centre-Sud | 0.59 |
| Africa | West Africa | Burkina Faso | Est | 0.65 |
| Africa | West Africa | Burkina Faso | Hauts-Bassins | 0.54 |
| Africa | West Africa | Burkina Faso | Nord | 0.57 |
| Africa | West Africa | Burkina Faso | Plateau Central | 0.59 |
| Africa | West Africa | Burkina Faso | Sahel | 0.72 |
| Africa | West Africa | Burkina Faso | Sud-Ouest | 0.67 |
| Africa | West Africa | Cote d'Ivoire | Abidjan | 0.74 |
| Africa | West Africa | Cote d'Ivoire | Central | 0.57 |
| Africa | West Africa | Cote d'Ivoire | East Central | 0.63 |
| Africa | West Africa | Cote d'Ivoire | North | 0.55 |
| Africa | West Africa | Cote d'Ivoire | North Central | 0.58 |
| Africa | West Africa | Cote d'Ivoire | Northeast | 0.55 |
| Africa | West Africa | Cote d'Ivoire | Northwest | 0.58 |
| Africa | West Africa | Cote d'Ivoire | South | 0.57 |
| Africa | West Africa | Cote d'Ivoire | Southwest | 0.60 |
| Africa | West Africa | Cote d'Ivoire | West | 0.56 |
| Africa | West Africa | Cote d'Ivoire | West Central | 0.59 |
| Africa | West Africa | Guinea | Boke | 0.64 |
| Africa | West Africa | Guinea | Conakry | 0.64 |
| Africa | West Africa | Guinea | Faranah | 0.63 |
| Africa | West Africa | Guinea | Kankan | 0.62 |
| Africa | West Africa | Guinea | Kindia | 0.72 |
| Africa | West Africa | Guinea | Labe | 0.69 |
| Africa | West Africa | Guinea | Mamou | 0.69 |
| Africa | West Africa | Guinea | Nzerekore | 0.60 |
| Africa | West Africa | Liberia | Monrovia | 0.46 |
| Africa | West Africa | Liberia | North Central | 0.72 |
| Africa | West Africa | Liberia | North Western | 0.76 |
| Africa | West Africa | Liberia | South Central | 0.66 |
| Africa | West Africa | Liberia | South Eastern A | 0.68 |
| Africa | West Africa | Liberia | South Eastern B | 0.72 |
| Africa | West Africa | Niger | Agadez | 0.53 |
| Africa | West Africa | Niger | Diffa | 0.50 |
| Africa | West Africa | Niger | Dosso | 0.55 |
| Africa | West Africa | Niger | Maradi | 0.51 |
| Africa | West Africa | Niger | Niamey | 0.56 |
| Africa | West Africa | Niger | Tahoua | 0.53 |
| Africa | West Africa | Niger | Tillaberi | 0.59 |
| Africa | West Africa | Niger | Zinder | 0.55 |
| Africa | West Africa | Nigeria | North Central | 0.70 |
| Africa | West Africa | Nigeria | North East | 0.65 |
| Africa | West Africa | Nigeria | North West | 0.61 |
| Africa | West Africa | Nigeria | South East | 0.66 |
| Africa | West Africa | Nigeria | South South | 0.64 |
| Africa | West Africa | Nigeria | South West | 0.52 |
| Africa | West Africa | Senegal | Dakar | 0.67 |
| Africa | West Africa | Senegal | Diourbel | 0.55 |
| Africa | West Africa | Senegal | Fatick | 0.54 |
| Africa | West Africa | Senegal | Kaffrine | 0.49 |
| Africa | West Africa | Senegal | Kaolack | 0.50 |
| Africa | West Africa | Senegal | Kedougou | 0.60 |
| Africa | West Africa | Senegal | Kolda | 0.60 |
| Africa | West Africa | Senegal | Louga | 0.52 |
| Africa | West Africa | Senegal | Matam | 0.54 |
| Africa | West Africa | Senegal | Saint-Louis | 0.57 |
| Africa | West Africa | Senegal | Sedhiou | 0.60 |
| Africa | West Africa | Senegal | Tambacounda | 0.56 |
| Africa | West Africa | Senegal | Thies | 0.56 |
| Africa | West Africa | Senegal | Ziguinchor | 0.58 |
| America | Latin America | Colombia | Amazonas | 0.52 |
| America | Latin America | Colombia | Antioquia | 0.62 |
| America | Latin America | Colombia | Arauca | 0.59 |
| America | Latin America | Colombia | Atlantico | 0.64 |
| America | Latin America | Colombia | Bogota | 0.70 |
| America | Latin America | Colombia | Bolivar | 0.64 |
| America | Latin America | Colombia | Boyaca | 0.62 |
| America | Latin America | Colombia | Caldas | 0.64 |
| America | Latin America | Colombia | Caqueta | 0.66 |
| America | Latin America | Colombia | Casanare | 0.58 |
| America | Latin America | Colombia | Cauca | 0.64 |
| America | Latin America | Colombia | Cesar | 0.63 |
| America | Latin America | Colombia | Choco | 0.64 |
| America | Latin America | Colombia | Cordoba | 0.59 |
| America | Latin America | Colombia | Cundinamarca | 0.63 |
| America | Latin America | Colombia | Guainia | 0.58 |
| America | Latin America | Colombia | Guaviare | 0.56 |
| America | Latin America | Colombia | Huila | 0.65 |
| America | Latin America | Colombia | La Guajira | 0.58 |
| America | Latin America | Colombia | Magdalena | 0.59 |
| America | Latin America | Colombia | Meta | 0.53 |
| America | Latin America | Colombia | Narino | 0.64 |
| America | Latin America | Colombia | Norte de Santander | 0.64 |
| America | Latin America | Colombia | Putumayo | 0.58 |
| America | Latin America | Colombia | Quindio | 0.66 |
| America | Latin America | Colombia | Risaralda | 0.65 |
| America | Latin America | Colombia | San Andres and Providencia | 0.42 |
| America | Latin America | Colombia | Santander | 0.68 |
| America | Latin America | Colombia | Sucre | 0.59 |
| America | Latin America | Colombia | Tolima | 0.63 |
| America | Latin America | Colombia | Valle | 0.63 |
| America | Latin America | Colombia | Vaupes | 0.56 |
| America | Latin America | Colombia | Vichada | 0.58 |
| America | Latin America | Honduras | Atlantida | 0.50 |
| America | Latin America | Honduras | Choluteca | 0.73 |
| America | Latin America | Honduras | Colon | 0.50 |
| America | Latin America | Honduras | Comayagua | 0.54 |
| America | Latin America | Honduras | Copan | 0.57 |
| America | Latin America | Honduras | El Paraiso | 0.76 |
| America | Latin America | Honduras | Gracias a Dios | 1.00 |
| America | Latin America | Honduras | Intibuca | 0.70 |
| America | Latin America | Honduras | Islas de la Bahia | 0.28 |
| America | Latin America | Honduras | La Paz | 0.64 |
| America | Latin America | Honduras | Lempira | 0.71 |
| America | Latin America | Honduras | Ocotepeque | 0.59 |
| America | Latin America | Honduras | Olancho | 0.64 |
| America | Latin America | Honduras | Resto Cortes | 0.47 |
| America | Latin America | Honduras | Resto Francisco Morazan | 0.62 |
| America | Latin America | Honduras | Santa Barbara | 0.57 |
| America | Latin America | Honduras | Valle | 0.67 |
| America | Latin America | Honduras | Yoro | 0.52 |
| Asia | Central Asia | Kyrgyz Republic | Batken | 0.60 |
| Asia | Central Asia | Kyrgyz Republic | Bishkek City | 0.84 |
| Asia | Central Asia | Kyrgyz Republic | Chui | 0.60 |
| Asia | Central Asia | Kyrgyz Republic | Djalal-Abad | 0.54 |
| Asia | Central Asia | Kyrgyz Republic | Issyk-Kul | 0.56 |
| Asia | Central Asia | Kyrgyz Republic | Naryn | 0.43 |
| Asia | Central Asia | Kyrgyz Republic | Osh City | 0.80 |
| Asia | Central Asia | Kyrgyz Republic | Osh Oblast | 0.64 |
| Asia | Central Asia | Kyrgyz Republic | Talas | 0.56 |
| Asia | Central Asia | Tajikistan | DRS | 0.62 |
| Asia | Central Asia | Tajikistan | Dushanbe | 0.84 |
| Asia | Central Asia | Tajikistan | GBAO | 0.63 |
| Asia | Central Asia | Tajikistan | Khatlon | 0.64 |
| Asia | Central Asia | Tajikistan | Sughd | 0.51 |
| Asia | South Asia | Bangladesh | Barisal | 0.62 |
| Asia | South Asia | Bangladesh | Chittagong | 0.47 |
| Asia | South Asia | Bangladesh | Dhaka | 0.31 |
| Asia | South Asia | Bangladesh | Khulna | 0.62 |
| Asia | South Asia | Bangladesh | Rajshahi | 0.60 |
| Asia | South Asia | Bangladesh | Rangpur | 0.60 |
| Asia | South Asia | Bangladesh | Sylhet | 0.61 |
| Asia | South Asia | Nepal | Central Hill | 0.53 |
| Asia | South Asia | Nepal | Central Mountain | 0.47 |
| Asia | South Asia | Nepal | Central Terai | 0.57 |
| Asia | South Asia | Nepal | Eastern Hill | 0.55 |
| Asia | South Asia | Nepal | Eastern Mountain | 0.44 |
| Asia | South Asia | Nepal | Eastern Terai | 0.58 |
| Asia | South Asia | Nepal | Far Western Hill | 0.45 |
| Asia | South Asia | Nepal | Far Western Terai | 0.60 |
| Asia | South Asia | Nepal | Far/Mid/Western Mountain | 0.43 |
| Asia | South Asia | Nepal | Mid Western Hill | 0.49 |
| Asia | South Asia | Nepal | Mid Western Terai | 0.54 |
| Asia | South Asia | Nepal | Western Hill | 0.45 |
| Asia | South Asia | Nepal | Western Terai | 0.57 |
| Asia | South-East Asia | Indonesia | Aceh | 0.69 |
| Asia | South-East Asia | Indonesia | Bali | 0.72 |
| Asia | South-East Asia | Indonesia | Bangka Belitung | 0.56 |
| Asia | South-East Asia | Indonesia | Banten | 0.60 |
| Asia | South-East Asia | Indonesia | Bengkulu | 0.68 |
| Asia | South-East Asia | Indonesia | Central Java | 0.72 |
| Asia | South-East Asia | Indonesia | Central Kalimantan | 0.72 |
| Asia | South-East Asia | Indonesia | Central Sulawesi | 0.79 |
| Asia | South-East Asia | Indonesia | DI Yogyakarta | 0.64 |
| Asia | South-East Asia | Indonesia | DKI Jakarta | 0.70 |
| Asia | South-East Asia | Indonesia | East Java | 0.67 |
| Asia | South-East Asia | Indonesia | East Kalimantan | 0.60 |
| Asia | South-East Asia | Indonesia | East Nusa Tenggara | 0.96 |
| Asia | South-East Asia | Indonesia | Gorontalo | 0.65 |
| Asia | South-East Asia | Indonesia | Jambi | 0.65 |
| Asia | South-East Asia | Indonesia | Lampung | 0.70 |
| Asia | South-East Asia | Indonesia | Maluku | 0.78 |
| Asia | South-East Asia | Indonesia | North Maluku | 0.66 |
| Asia | South-East Asia | Indonesia | North Sulawesi | 0.77 |
| Asia | South-East Asia | Indonesia | North Sumatera | 0.67 |
| Asia | South-East Asia | Indonesia | Papua | 0.81 |
| Asia | South-East Asia | Indonesia | Riau | 0.62 |
| Asia | South-East Asia | Indonesia | Riau Islands | 0.64 |
| Asia | South-East Asia | Indonesia | South Kalimantan | 0.68 |
| Asia | South-East Asia | Indonesia | South Sulawesi | 0.68 |
| Asia | South-East Asia | Indonesia | South Sumatera | 0.61 |
| Asia | South-East Asia | Indonesia | Southeast Sulawesi | 0.85 |
| Asia | South-East Asia | Indonesia | West Java | 0.67 |
| Asia | South-East Asia | Indonesia | West Kalimantan | 0.74 |
| Asia | South-East Asia | Indonesia | West Nusa Tenggara | 0.70 |
| Asia | South-East Asia | Indonesia | West Papua | 0.73 |
| Asia | South-East Asia | Indonesia | West Sulawesi | 0.84 |
| Asia | South-East Asia | Indonesia | West Sumatera | 0.69 |
| Asia | South-East Asia | Timor-Leste | Aileu | 0.50 |
| Asia | South-East Asia | Timor-Leste | Ainaro | 0.57 |
| Asia | South-East Asia | Timor-Leste | Baucau | 0.55 |
| Asia | South-East Asia | Timor-Leste | Bobonaro | 0.46 |
| Asia | South-East Asia | Timor-Leste | Covalima | 0.50 |
| Asia | South-East Asia | Timor-Leste | Dili | 0.50 |
| Asia | South-East Asia | Timor-Leste | Ermera | 0.53 |
| Asia | South-East Asia | Timor-Leste | Lautem | 0.48 |
| Asia | South-East Asia | Timor-Leste | Liquica | 0.58 |
| Asia | South-East Asia | Timor-Leste | Manatuto | 0.51 |
| Asia | South-East Asia | Timor-Leste | Manufahi | 0.54 |
| Asia | South-East Asia | Timor-Leste | Oecusse | 0.48 |
| Asia | South-East Asia | Timor-Leste | Viqueque | 0.54 |
| Asia | West Asia | Armenia | Aragatsotn | 0.61 |
| Asia | West Asia | Armenia | Ararat | 0.62 |
| Asia | West Asia | Armenia | Armavir | 0.63 |
| Asia | West Asia | Armenia | Gegharkunik | 0.59 |
| Asia | West Asia | Armenia | Kotayk | 0.63 |
| Asia | West Asia | Armenia | Lori | 0.61 |
| Asia | West Asia | Armenia | Shirak | 0.57 |
| Asia | West Asia | Armenia | Syunik | 0.60 |
| Asia | West Asia | Armenia | Tavush | 0.60 |
| Asia | West Asia | Armenia | Vayots Dzor | 0.56 |
| Asia | West Asia | Armenia | Yerevan | 0.82 |
| Asia | West Asia | Jordan | Ajloun | 0.57 |
| Asia | West Asia | Jordan | Amman | 0.44 |
| Asia | West Asia | Jordan | Aqaba | 0.57 |
| Asia | West Asia | Jordan | Balqa | 0.61 |
| Asia | West Asia | Jordan | Irbid | 0.59 |
| Asia | West Asia | Jordan | Jarash | 0.52 |
| Asia | West Asia | Jordan | Karak | 0.46 |
| Asia | West Asia | Jordan | Ma'an | 0.45 |
| Asia | West Asia | Jordan | Madaba | 0.44 |
| Asia | West Asia | Jordan | Mafraq | 0.47 |
| Asia | West Asia | Jordan | Tafiela | 0.54 |
| Asia | West Asia | Jordan | Zarqa | 0.47 |
| Caribbean | Caribbean | Haiti | Artibonite | 0.57 |
| Caribbean | Caribbean | Haiti | Central | 0.54 |
| Caribbean | Caribbean | Haiti | Grande-Anse | 0.59 |
| Caribbean | Caribbean | Haiti | Metropolitan Area | 0.52 |
| Caribbean | Caribbean | Haiti | Nippes | 0.49 |
| Caribbean | Caribbean | Haiti | North | 0.49 |
| Caribbean | Caribbean | Haiti | Northeast | 0.52 |
| Caribbean | Caribbean | Haiti | Northwest | 0.54 |
| Caribbean | Caribbean | Haiti | South | 0.54 |
| Caribbean | Caribbean | Haiti | Southeast | 0.54 |

(b) Group 2 (2005 – 2009)

| **Continent** | **Region** | **Country** | **Sub-national boundary (by DHS)** | **TRF index** |
| --- | --- | --- | --- | --- |
| Africa | Central Africa | Congo, Dem. Rep | Bandundu | 0.79 |
| Africa | Central Africa | Congo, Dem. Rep | Bas-Congo | 0.68 |
| Africa | Central Africa | Congo, Dem. Rep | Equateur | 0.74 |
| Africa | Central Africa | Congo, Dem. Rep | Kasai Occident | 0.80 |
| Africa | Central Africa | Congo, Dem. Rep | Kasai Oriental | 0.51 |
| Africa | Central Africa | Congo, Dem. Rep | Katanga | 0.76 |
| Africa | Central Africa | Congo, Dem. Rep | Kinshasa | 0.46 |
| Africa | Central Africa | Congo, Dem. Rep | Maniema | 0.73 |
| Africa | Central Africa | Congo, Dem. Rep | Nord-Kivu | 0.52 |
| Africa | Central Africa | Congo, Dem. Rep | Orientale | 0.66 |
| Africa | Central Africa | Congo, Dem. Rep | Sud-Kivu | 0.67 |
| Africa | Central Africa | Sao Tome and Principe | Central | 0.46 |
| Africa | Central Africa | Sao Tome and Principe | North | 0.34 |
| Africa | Central Africa | Sao Tome and Principe | Principe | 0.75 |
| Africa | Central Africa | Sao Tome and Principe | South | 0.33 |
| Africa | East Africa | Kenya | Central | 0.75 |
| Africa | East Africa | Kenya | Coast | 0.50 |
| Africa | East Africa | Kenya | Eastern | 0.81 |
| Africa | East Africa | Kenya | Nairobi Area | 0.76 |
| Africa | East Africa | Kenya | North-Eastern | 0.77 |
| Africa | East Africa | Kenya | Nyanza | 0.81 |
| Africa | East Africa | Kenya | Rift Valley | 0.83 |
| Africa | East Africa | Kenya | Western | 0.72 |
| Africa | East Africa | Zambia | Central | 0.71 |
| Africa | East Africa | Zambia | Copperbelt | 0.64 |
| Africa | East Africa | Zambia | Eastern | 0.80 |
| Africa | East Africa | Zambia | Luapula | 0.96 |
| Africa | East Africa | Zambia | Lusaka | 0.40 |
| Africa | East Africa | Zambia | North-Western | 0.74 |
| Africa | East Africa | Zambia | Northern | 1.00 |
| Africa | East Africa | Zambia | Southern | 0.79 |
| Africa | East Africa | Zambia | Western | 0.71 |
| Africa | North Africa | Egypt | Frontier Governorates | 0.63 |
| Africa | North Africa | Egypt | Lower Egypt | 0.86 |
| Africa | North Africa | Egypt | Upper Egypt | 0.67 |
| Africa | North Africa | Egypt | Urban Governorates | 0.75 |
| Africa | South Africa | Lesotho | Berea | 0.46 |
| Africa | South Africa | Lesotho | Butha-Buthe | 0.37 |
| Africa | South Africa | Lesotho | Leribe | 0.46 |
| Africa | South Africa | Lesotho | Mafeteng | 0.40 |
| Africa | South Africa | Lesotho | Maseru | 0.44 |
| Africa | South Africa | Lesotho | Mohale's Hoek | 0.43 |
| Africa | South Africa | Lesotho | Mokhotlong | 0.42 |
| Africa | South Africa | Lesotho | Qacha's Nek | 0.34 |
| Africa | South Africa | Lesotho | Quthing | 0.41 |
| Africa | South Africa | Lesotho | Thaba-Tseka | 0.47 |
| Africa | South Africa | Namibia | Caprivi | 0.54 |
| Africa | South Africa | Namibia | Erongo | 0.49 |
| Africa | South Africa | Namibia | Hardap | 0.50 |
| Africa | South Africa | Namibia | Karas | 0.54 |
| Africa | South Africa | Namibia | Kavango | 0.58 |
| Africa | South Africa | Namibia | Khomas | 0.54 |
| Africa | South Africa | Namibia | Kunene | 0.58 |
| Africa | South Africa | Namibia | Ohangwena | 0.53 |
| Africa | South Africa | Namibia | Omaheke | 0.57 |
| Africa | South Africa | Namibia | Omusati | 0.68 |
| Africa | South Africa | Namibia | Oshana | 0.33 |
| Africa | South Africa | Namibia | Oshikoto | 0.45 |
| Africa | South Africa | Namibia | Otjozondjupa | 0.49 |
| Africa | South Africa | Swaziland | Hhohho | 0.65 |
| Africa | South Africa | Swaziland | Lubombo | 0.76 |
| Africa | South Africa | Swaziland | Manzini | 0.69 |
| Africa | South Africa | Swaziland | Shiselweni | 0.76 |
| Africa | West Africa | Ghana | Ashanti | 0.54 |
| Africa | West Africa | Ghana | Brong-Ahafo | 0.63 |
| Africa | West Africa | Ghana | Central | 0.45 |
| Africa | West Africa | Ghana | Eastern | 0.59 |
| Africa | West Africa | Ghana | Greater Accra | 0.50 |
| Africa | West Africa | Ghana | Northern | 0.70 |
| Africa | West Africa | Ghana | Upper East | 0.60 |
| Africa | West Africa | Ghana | Upper West | 0.60 |
| Africa | West Africa | Ghana | Volta | 0.57 |
| Africa | West Africa | Ghana | Western | 0.64 |
| Africa | West Africa | Sierra Leone | Eastern | 0.80 |
| Africa | West Africa | Sierra Leone | Northern | 0.94 |
| Africa | West Africa | Sierra Leone | Southern | 0.87 |
| Africa | West Africa | Sierra Leone | Western | 0.43 |
| America | Latin America | Guyana | Region 1 | 0.75 |
| America | Latin America | Guyana | Region 10 | 0.63 |
| America | Latin America | Guyana | Region 2 | 0.67 |
| America | Latin America | Guyana | Region 3 | 0.60 |
| America | Latin America | Guyana | Region 4 | 0.43 |
| America | Latin America | Guyana | Region 5 | 0.57 |
| America | Latin America | Guyana | Region 6 | 0.56 |
| America | Latin America | Guyana | Region 7 | 0.76 |
| America | Latin America | Guyana | Region 8 | 0.79 |
| America | Latin America | Guyana | Region 9 | 0.65 |
| America | Latin America | Peru | Amazonas | 0.76 |
| America | Latin America | Peru | Ancash | 0.71 |
| America | Latin America | Peru | Apurimac | 0.83 |
| America | Latin America | Peru | Arequipa | 0.62 |
| America | Latin America | Peru | Ayacucho | 0.74 |
| America | Latin America | Peru | Cajamarca | 0.81 |
| America | Latin America | Peru | Cusco | 0.85 |
| America | Latin America | Peru | Huancavelica | 0.84 |
| America | Latin America | Peru | Huanuco | 0.87 |
| America | Latin America | Peru | Ica | 0.69 |
| America | Latin America | Peru | Junin | 0.76 |
| America | Latin America | Peru | La Libertad | 0.73 |
| America | Latin America | Peru | Lambayaque | 0.77 |
| America | Latin America | Peru | Lima | 0.67 |
| America | Latin America | Peru | Loreto | 0.87 |
| America | Latin America | Peru | Madre de Dios | 0.77 |
| America | Latin America | Peru | Moquegua | 0.65 |
| America | Latin America | Peru | Pasco | 0.76 |
| America | Latin America | Peru | Piura | 0.74 |
| America | Latin America | Peru | Puno | 0.92 |
| America | Latin America | Peru | San Martin | 0.82 |
| America | Latin America | Peru | Tacna | 0.67 |
| America | Latin America | Peru | Tumbes | 0.66 |
| America | Latin America | Peru | Ucayali | 0.91 |
| Asia | South Asia | India | Andhra Pradesh | 0.44 |
| Asia | South Asia | India | Arunachal Pradesh | 0.42 |
| Asia | South Asia | India | Assam | 0.65 |
| Asia | South Asia | India | Bihar | 0.63 |
| Asia | South Asia | India | Chhattisgarh | 0.62 |
| Asia | South Asia | India | Delhi | 0.72 |
| Asia | South Asia | India | Goa | 0.67 |
| Asia | South Asia | India | Gujarat | 0.61 |
| Asia | South Asia | India | Haryana | 0.60 |
| Asia | South Asia | India | Himachal Pradesh | 0.58 |
| Asia | South Asia | India | Jammu and Kashmir | 0.41 |
| Asia | South Asia | India | Jharkhard | 0.67 |
| Asia | South Asia | India | Karnataka | 0.51 |
| Asia | South Asia | India | Kerala | 0.63 |
| Asia | South Asia | India | Madhya Pradesh | 0.58 |
| Asia | South Asia | India | Maharashtra | 0.54 |
| Asia | South Asia | India | Manipur | 0.70 |
| Asia | South Asia | India | Meghalaya | 0.49 |
| Asia | South Asia | India | Mizoram | 0.58 |
| Asia | South Asia | India | Nagaland | 0.43 |
| Asia | South Asia | India | Orissa | 0.65 |
| Asia | South Asia | India | Punjab | 0.65 |
| Asia | South Asia | India | Rajasthan | 0.55 |
| Asia | South Asia | India | Sikkim | 0.61 |
| Asia | South Asia | India | Tamil Nadu | 0.00 |
| Asia | South Asia | India | Tripura | 0.55 |
| Asia | South Asia | India | Uttar Pradesh | 0.60 |
| Asia | South Asia | India | Uttaranchal | 0.57 |
| Asia | South Asia | India | West Bengal | 0.59 |
| Asia | South Asia | Maldives | Central | 0.53 |
| Asia | South Asia | Maldives | Male | 0.78 |
| Asia | South Asia | Maldives | North | 0.65 |
| Asia | South Asia | Maldives | North Central | 0.59 |
| Asia | South Asia | Maldives | South | 0.65 |
| Asia | South Asia | Maldives | South Central | 0.63 |
| Asia | South Asia | Pakistan | Balochistan | 0.76 |
| Asia | South Asia | Pakistan | NW Frontier Province | 0.59 |
| Asia | South Asia | Pakistan | Punjab | 0.93 |
| Asia | South Asia | Pakistan | Sindh | 0.99 |
| Asia | South-East Asia | Philippines | ARMM | 0.65 |
| Asia | South-East Asia | Philippines | Bicol | 0.52 |
| Asia | South-East Asia | Philippines | Cagayan Valley | 0.55 |
| Asia | South-East Asia | Philippines | Calabrazon | 0.54 |
| Asia | South-East Asia | Philippines | Caraga | 0.46 |
| Asia | South-East Asia | Philippines | Central Luzon | 0.56 |
| Asia | South-East Asia | Philippines | Central Visayas | 0.46 |
| Asia | South-East Asia | Philippines | Cordillera Administrative Region | 0.50 |
| Asia | South-East Asia | Philippines | Davao | 0.56 |
| Asia | South-East Asia | Philippines | Eastern Visayas | 0.43 |
| Asia | South-East Asia | Philippines | Ilocos | 0.57 |
| Asia | South-East Asia | Philippines | Mimaropa | 0.60 |
| Asia | South-East Asia | Philippines | National Capital Region | 0.86 |
| Asia | South-East Asia | Philippines | Northern Mindanao | 0.53 |
| Asia | South-East Asia | Philippines | Soccsksargen | 0.58 |
| Asia | South-East Asia | Philippines | Western Visayas | 0.50 |
| Asia | South-East Asia | Philippines | Zamboanga Peninsula | 0.52 |
| Asia | South-East Asia | Vietnam | Central | 0.79 |
| Asia | South-East Asia | Vietnam | North | 0.87 |
| Asia | South-East Asia | Vietnam | South | 0.83 |
| Asia | West Asia | Azerbaijan | Absheron | 0.66 |
| Asia | West Asia | Azerbaijan | Aran | 0.86 |
| Asia | West Asia | Azerbaijan | Baku | 0.82 |
| Asia | West Asia | Azerbaijan | Daghligh Shirvan | 0.49 |
| Asia | West Asia | Azerbaijan | Ganja Gazakh | 0.61 |
| Asia | West Asia | Azerbaijan | Guba Khachmaz | 0.21 |
| Asia | West Asia | Azerbaijan | Lankaran | 0.62 |
| Asia | West Asia | Azerbaijan | Shaki Zaqatala | 0.66 |
| Asia | West Asia | Azerbaijan | Yukhari Garabakh | 0.55 |
| Caribbean | Caribbean | Dominican Republic | Azua | 0.55 |
| Caribbean | Caribbean | Dominican Republic | Bahoruco | 0.61 |
| Caribbean | Caribbean | Dominican Republic | Barahona | 0.56 |
| Caribbean | Caribbean | Dominican Republic | Dajabon | 0.54 |
| Caribbean | Caribbean | Dominican Republic | Distrito Nacional | 0.86 |
| Caribbean | Caribbean | Dominican Republic | Duarte | 0.48 |
| Caribbean | Caribbean | Dominican Republic | El Seibo | 0.56 |
| Caribbean | Caribbean | Dominican Republic | Elias Pina | 0.69 |
| Caribbean | Caribbean | Dominican Republic | Espaillat | 0.52 |
| Caribbean | Caribbean | Dominican Republic | Hato Mayor | 0.46 |
| Caribbean | Caribbean | Dominican Republic | Independencia | 0.58 |
| Caribbean | Caribbean | Dominican Republic | La Altagracia | 0.36 |
| Caribbean | Caribbean | Dominican Republic | La Romana | 0.42 |
| Caribbean | Caribbean | Dominican Republic | La Vega | 0.53 |
| Caribbean | Caribbean | Dominican Republic | Maria Trinidad Sanchez | 0.51 |
| Caribbean | Caribbean | Dominican Republic | Monsenor Nouel | 0.57 |
| Caribbean | Caribbean | Dominican Republic | Monte Cristi | 0.41 |
| Caribbean | Caribbean | Dominican Republic | Monte Plata | 0.60 |
| Caribbean | Caribbean | Dominican Republic | Pedernales | 0.53 |
| Caribbean | Caribbean | Dominican Republic | Peravia | 0.48 |
| Caribbean | Caribbean | Dominican Republic | Puerto Plata | 0.42 |
| Caribbean | Caribbean | Dominican Republic | Salcedo | 0.55 |
| Caribbean | Caribbean | Dominican Republic | Samana | 0.50 |
| Caribbean | Caribbean | Dominican Republic | San Cristobal | 0.56 |
| Caribbean | Caribbean | Dominican Republic | San Jose de Ocoa | 0.50 |
| Caribbean | Caribbean | Dominican Republic | San Juan | 0.61 |
| Caribbean | Caribbean | Dominican Republic | San Pedro de Macoris | 0.46 |
| Caribbean | Caribbean | Dominican Republic | Sanchez Ramirez | 0.48 |
| Caribbean | Caribbean | Dominican Republic | Santiago | 0.44 |
| Caribbean | Caribbean | Dominican Republic | Santiago Rodriguez | 0.50 |
| Caribbean | Caribbean | Dominican Republic | Santo Domingo | 0.56 |
| Caribbean | Caribbean | Dominican Republic | Valverde | 0.48 |
| Europe | East Europe | Moldova | Center | 0.65 |
| Europe | East Europe | Moldova | Chisinau | 0.76 |
| Europe | East Europe | Moldova | North | 0.65 |
| Europe | East Europe | Moldova | South | 0.63 |
| Europe | East Europe | Ukraine | Central | 0.63 |
| Europe | East Europe | Ukraine | East | 0.62 |
| Europe | East Europe | Ukraine | North | 0.64 |
| Europe | East Europe | Ukraine | South | 0.63 |
| Europe | East Europe | Ukraine | West | 0.65 |
| Europe | South-East Europe | Albania | Central | 0.52 |
| Europe | South-East Europe | Albania | Coastal | 0.37 |
| Europe | South-East Europe | Albania | Mountain | 0.15 |
| Europe | South-East Europe | Albania | Tirana | 0.95 |

(c) Group 3 (before 2005)

| **Continent** | **Region** | **Country** | **Sub-national boundary (by DHS)** | **TRF index** |
| --- | --- | --- | --- | --- |
| Africa | Central Africa | Central African Republic | Bangui | 0.02 |
| Africa | Central Africa | Central African Republic | Region 1 | 0.85 |
| Africa | Central Africa | Central African Republic | Region 2 | 0.87 |
| Africa | Central Africa | Central African Republic | Region 3 | 0.82 |
| Africa | Central Africa | Central African Republic | Region 4 | 0.73 |
| Africa | Central Africa | Central African Republic | Region 5 | 0.86 |
| Africa | Central Africa | Chad | Zone 1 | 0.28 |
| Africa | Central Africa | Chad | Zone 2 | 0.63 |
| Africa | Central Africa | Chad | Zone 3 | 0.82 |
| Africa | Central Africa | Chad | Zone 4 | 0.58 |
| Africa | Central Africa | Chad | Zone 5 | 0.51 |
| Africa | Central Africa | Chad | Zone 6 | 0.61 |
| Africa | Central Africa | Chad | Zone 7 | 0.64 |
| Africa | Central Africa | Chad | Zone 8 | 0.64 |
| Africa | North Africa | Morocco | Chaouia - Ouardigha | 0.45 |
| Africa | North Africa | Morocco | Doukkala - Abda | 0.40 |
| Africa | North Africa | Morocco | Fes - Boulemane | 0.43 |
| Africa | North Africa | Morocco | Gharb - Chrarda - Beni Hssen | 0.30 |
| Africa | North Africa | Morocco | Grand Casablanca | 0.55 |
| Africa | North Africa | Morocco | Guelmim - Es-Semara | 0.36 |
| Africa | North Africa | Morocco | Laayoune - Boujdour - Sakia El Hamra | 0.25 |
| Africa | North Africa | Morocco | Marrakech - Tensift - Al Haouz | 0.48 |
| Africa | North Africa | Morocco | Meknes - Tafilalet | 0.36 |
| Africa | North Africa | Morocco | Oriental | 0.41 |
| Africa | North Africa | Morocco | Rabat - Sale - Zemmour - Zaer | 0.40 |
| Africa | North Africa | Morocco | Souss - Massa - Draa | 0.38 |
| Africa | North Africa | Morocco | Tadla - Azilal | 0.47 |
| Africa | North Africa | Morocco | Tanger - Tetouan | 0.50 |
| Africa | North Africa | Morocco | Taza - Al Hoceima - Taounate | 0.53 |
| Africa | South Africa | South Africa | Eastern Cape | 0.61 |
| Africa | South Africa | South Africa | Free State | 0.32 |
| Africa | South Africa | South Africa | Gauteng | 0.57 |
| Africa | South Africa | South Africa | KwaZulu Natal | 0.49 |
| Africa | South Africa | South Africa | Mpumalanga | 0.30 |
| Africa | South Africa | South Africa | North West | 0.19 |
| Africa | South Africa | South Africa | Northern Cape | 0.42 |
| Africa | South Africa | South Africa | Northern Province | 0.19 |
| Africa | South Africa | South Africa | Western Cape | 0.43 |
| Africa | West Africa | Togo | Centrale | 0.63 |
| Africa | West Africa | Togo | Kara | 0.54 |
| Africa | West Africa | Togo | Lome | 0.73 |
| Africa | West Africa | Togo | Maritime | 0.29 |
| Africa | West Africa | Togo | Plateaux | 0.81 |
| Africa | West Africa | Togo | Savanes | 0.63 |
| America | Latin America | Brazil | Centro-Leste | 0.46 |
| America | Latin America | Brazil | Centro-Oeste | 0.42 |
| America | Latin America | Brazil | Nordeste | 0.47 |
| America | Latin America | Brazil | Norte | 0.41 |
| America | Latin America | Brazil | Rio de Janeiro | 0.54 |
| America | Latin America | Brazil | Sao Paulo | 0.44 |
| America | Latin America | Brazil | Sul | 0.45 |
| America | Latin America | Guatemala | Central | 0.26 |
| America | Latin America | Guatemala | Metropolitan | 0.13 |
| America | Latin America | Guatemala | North | 0.60 |
| America | Latin America | Guatemala | Northeast | 0.46 |
| America | Latin America | Guatemala | Northwest | 0.44 |
| America | Latin America | Guatemala | Peten | 0.57 |
| America | Latin America | Guatemala | Southeast | 0.39 |
| America | Latin America | Guatemala | Southwest | 0.43 |
| America | Latin America | Nicaragua | Boaco | 0.63 |
| America | Latin America | Nicaragua | Carazo | 0.49 |
| America | Latin America | Nicaragua | Chinadega | 0.51 |
| America | Latin America | Nicaragua | Chontales | 0.56 |
| America | Latin America | Nicaragua | Esteli | 0.54 |
| America | Latin America | Nicaragua | Granada | 0.48 |
| America | Latin America | Nicaragua | Jinotega | 0.70 |
| America | Latin America | Nicaragua | Leon | 0.50 |
| America | Latin America | Nicaragua | Madriz | 0.67 |
| America | Latin America | Nicaragua | Managua | 0.49 |
| America | Latin America | Nicaragua | Masaya | 0.57 |
| America | Latin America | Nicaragua | Matagalpa | 0.58 |
| America | Latin America | Nicaragua | New Segovia | 0.59 |
| America | Latin America | Nicaragua | North Atlantic Autonomous Region (RAAN) | 0.68 |
| America | Latin America | Nicaragua | Rio San Juan | 0.66 |
| America | Latin America | Nicaragua | Rivas | 0.48 |
| America | Latin America | Nicaragua | South Atlantic Autonomous Region (RAAS) | 0.66 |
| Asia | Central Asia | Kazakhstan | Almaty City | 1.00 |
| Asia | Central Asia | Kazakhstan | Central | 0.43 |
| Asia | Central Asia | Kazakhstan | East | 0.39 |
| Asia | Central Asia | Kazakhstan | North | 0.41 |
| Asia | Central Asia | Kazakhstan | South | 0.32 |
| Asia | Central Asia | Kazakhstan | West | 0.36 |
| Asia | West Asia | Yemen | North & West | 0.80 |
| Asia | West Asia | Yemen | South & East | 0.58 |
| Europe | South-East Europe | Turkey | Central | 0.30 |
| Europe | South-East Europe | Turkey | East | 0.46 |
| Europe | South-East Europe | Turkey | North | 0.34 |
| Europe | South-East Europe | Turkey | South | 0.38 |
| Europe | South-East Europe | Turkey | West | 0.00 |

**Appendix 5. TRF index in Bangladesh**

**A5. TRF index in Bangladesh**
