# Supplementary figures and images for: Geographical distribution of typhoid risk factors in low and middle income countries
Source: BMC Infect Dis. 2016 Dec 5;16:732. doi: 10.1186/s12879-016-2074-1 (PMC5139008; doi:10.1186/s12879-016-2074-1)

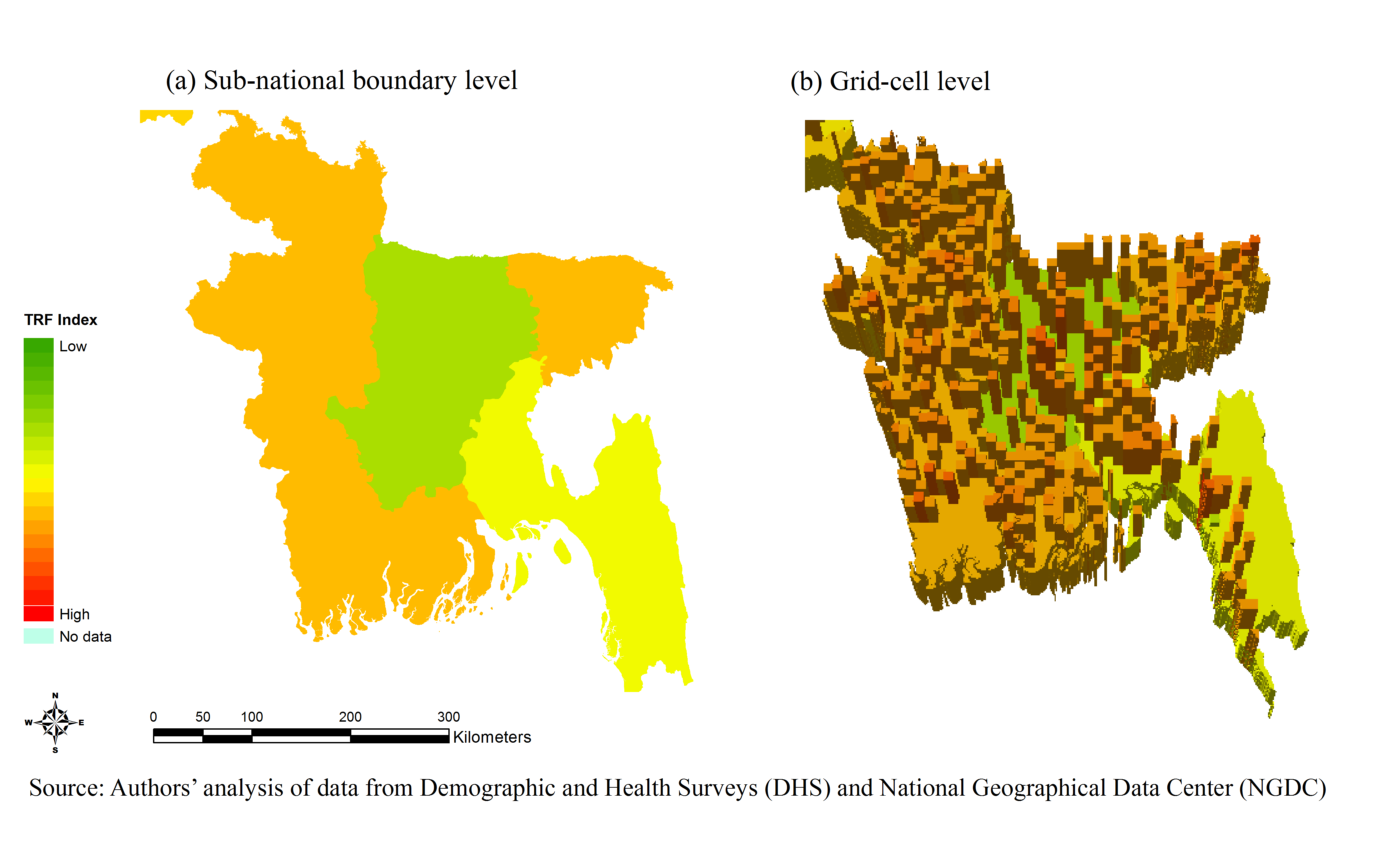

Supplement: Additional file 1: Appendix 1. — Overdispersion. Appendix 2. Full specification of regression outputs. Appendix 3. TRF index (type 5) by sub-national boundary. Appendix 4. Final TRF index values by sub-national boundary. Appendix 5. TRF index in Bangladesh. (ZIP 1995 kb) [file 12879_2016_2074_MOESM1_ESM.zip › A5R2.tif]

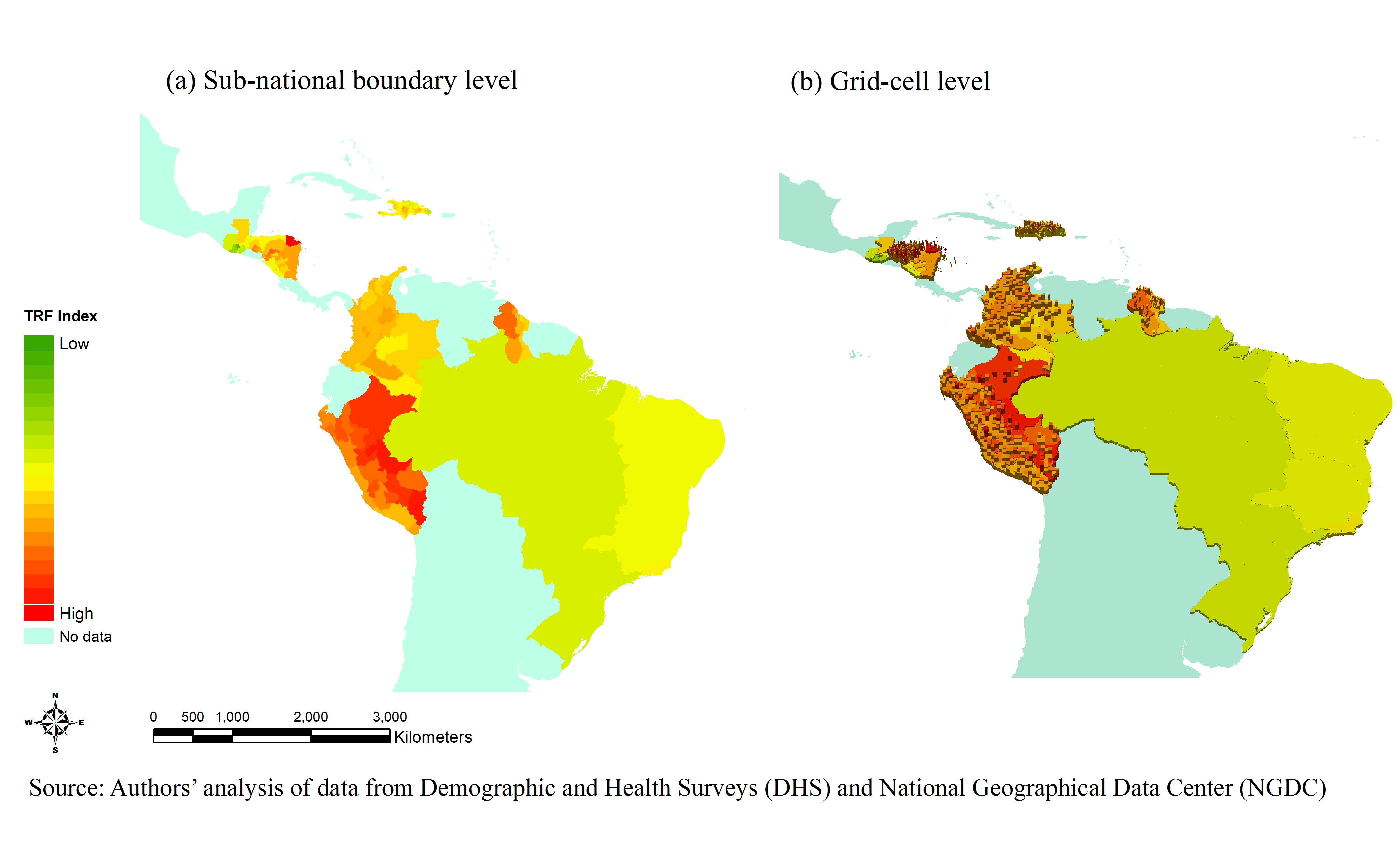

Supplement: Additional file 1: Appendix 1. — Overdispersion. Appendix 2. Full specification of regression outputs. Appendix 3. TRF index (type 5) by sub-national boundary. Appendix 4. Final TRF index values by sub-national boundary. Appendix 5. TRF index in Bangladesh. (ZIP 1995 kb) [file 12879_2016_2074_MOESM1_ESM.zip › A3-4R2.tif]

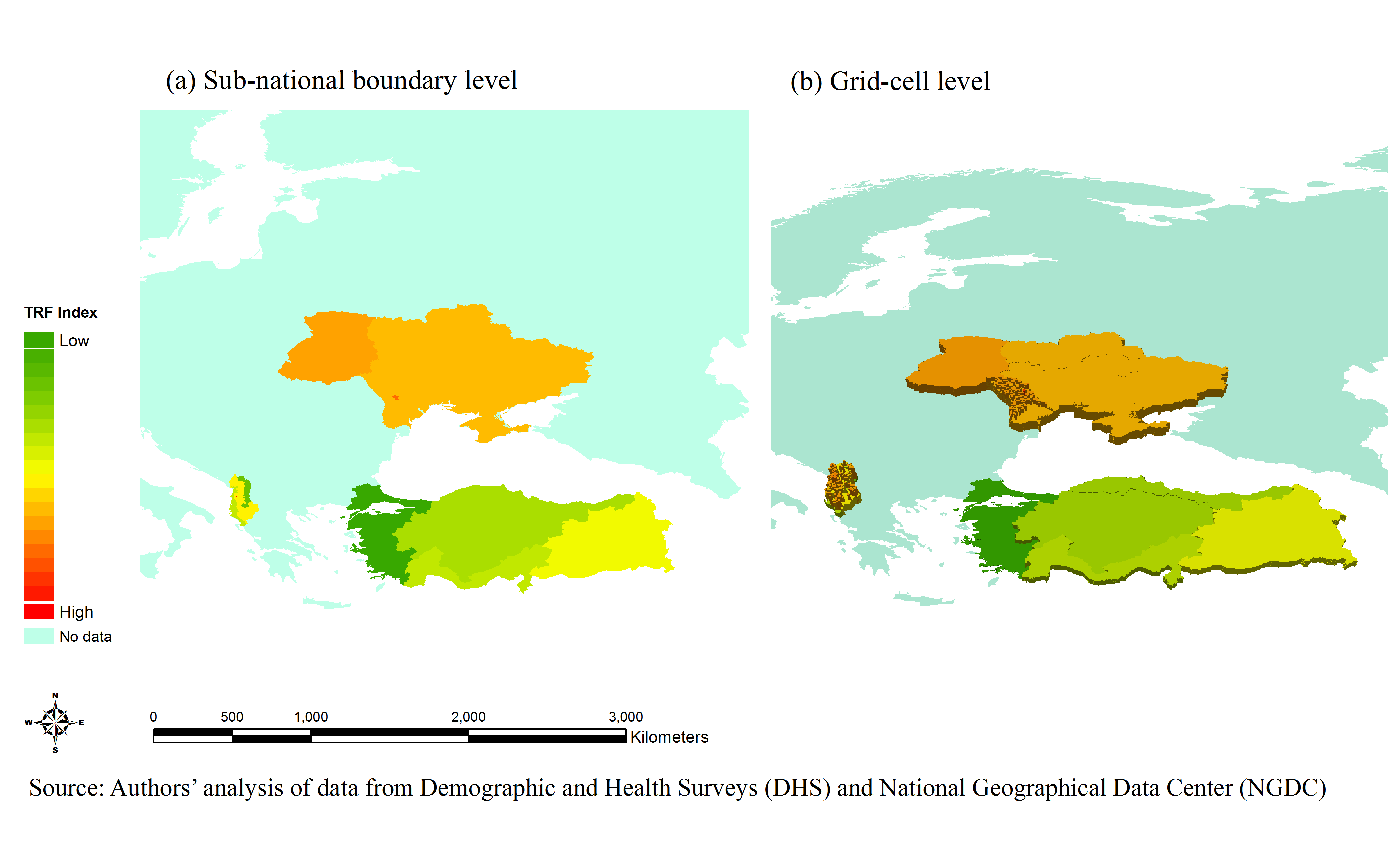

Supplement: Additional file 1: Appendix 1. — Overdispersion. Appendix 2. Full specification of regression outputs. Appendix 3. TRF index (type 5) by sub-national boundary. Appendix 4. Final TRF index values by sub-national boundary. Appendix 5. TRF index in Bangladesh. (ZIP 1995 kb) [file 12879_2016_2074_MOESM1_ESM.zip › A3-5R2.tif]

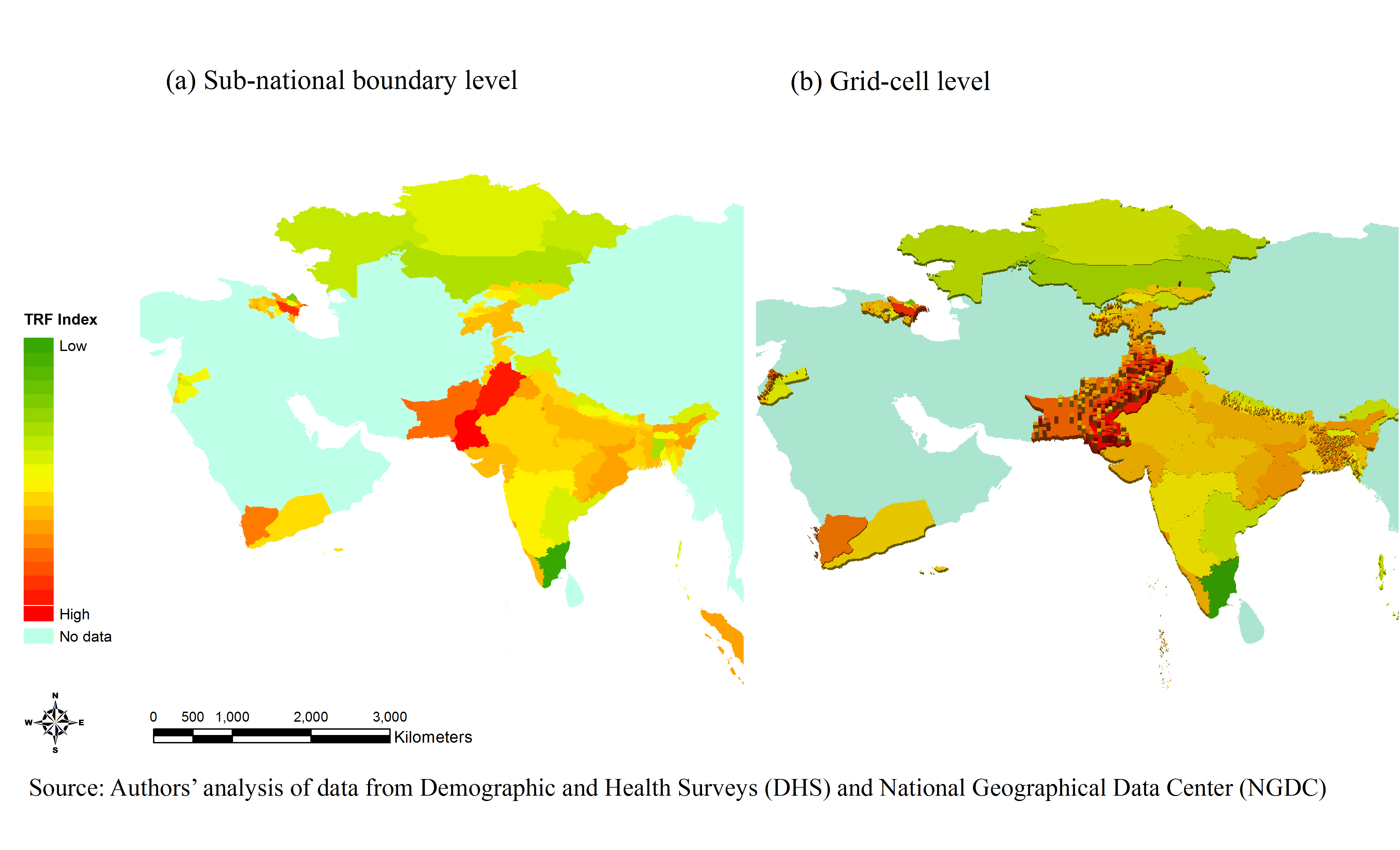

Supplement: Additional file 1: Appendix 1. — Overdispersion. Appendix 2. Full specification of regression outputs. Appendix 3. TRF index (type 5) by sub-national boundary. Appendix 4. Final TRF index values by sub-national boundary. Appendix 5. TRF index in Bangladesh. (ZIP 1995 kb) [file 12879_2016_2074_MOESM1_ESM.zip › A3-2R2.tif]

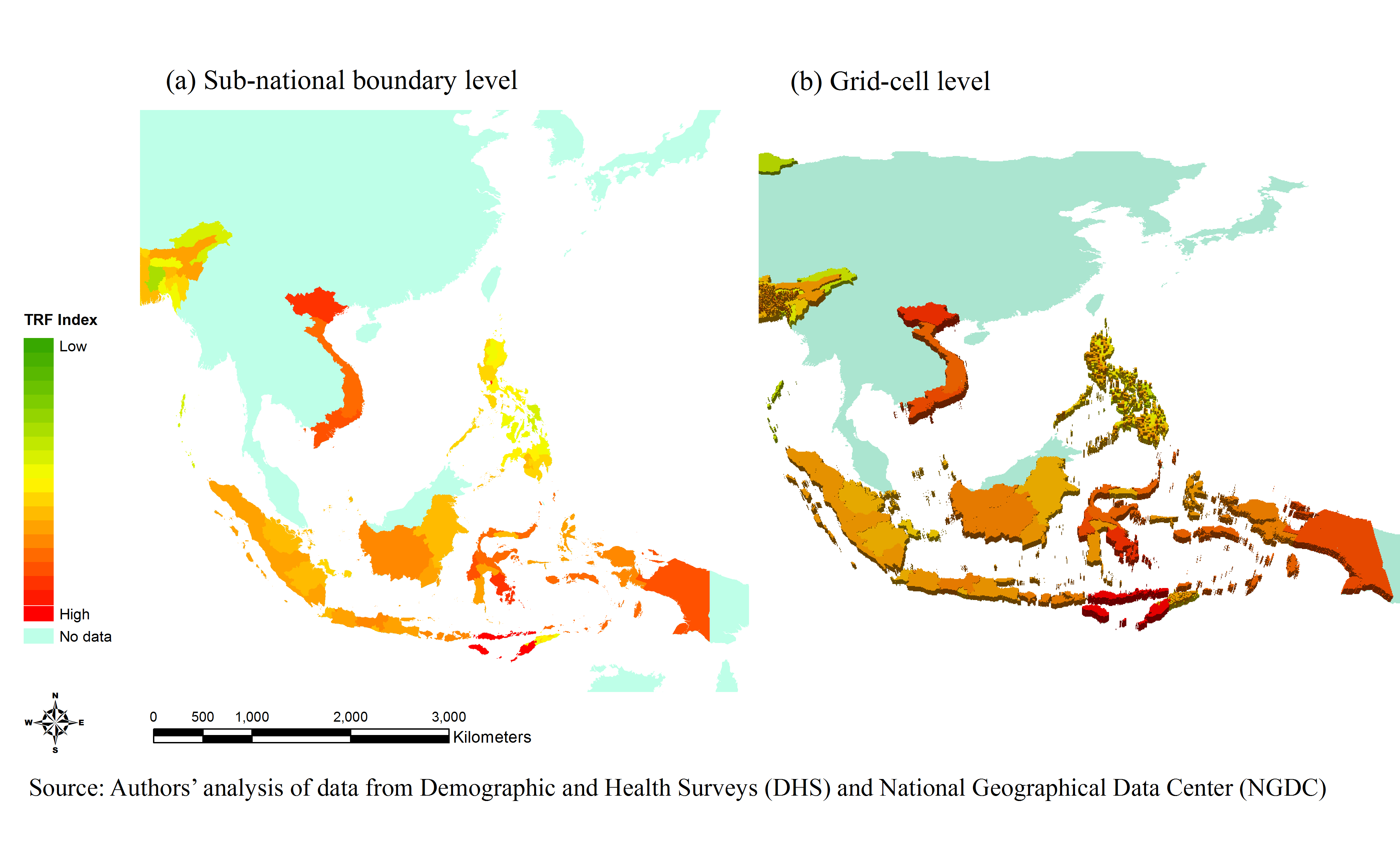

Supplement: Additional file 1: Appendix 1. — Overdispersion. Appendix 2. Full specification of regression outputs. Appendix 3. TRF index (type 5) by sub-national boundary. Appendix 4. Final TRF index values by sub-national boundary. Appendix 5. TRF index in Bangladesh. (ZIP 1995 kb) [file 12879_2016_2074_MOESM1_ESM.zip › A3-3R2.tif]

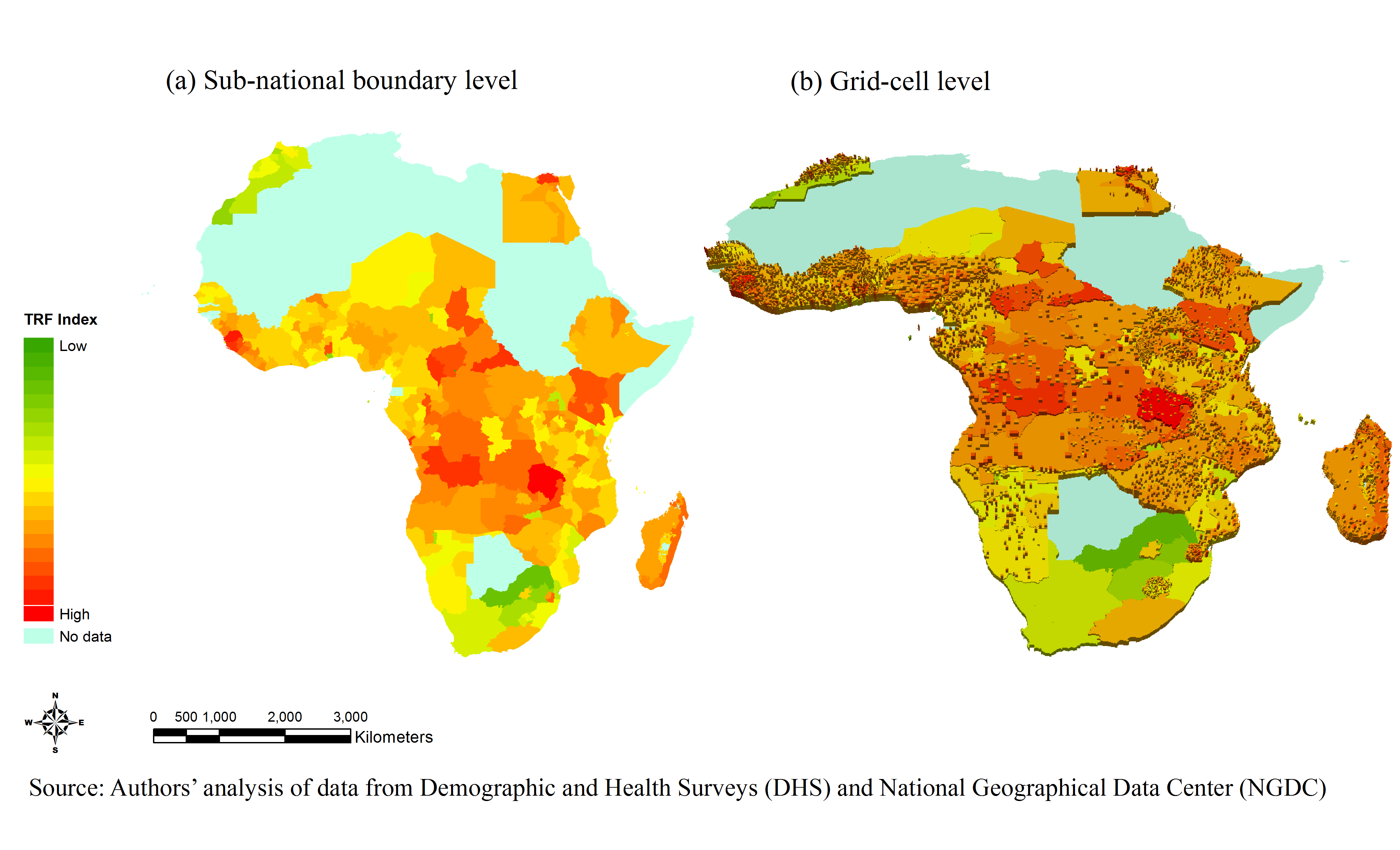

Supplement: Additional file 1: Appendix 1. — Overdispersion. Appendix 2. Full specification of regression outputs. Appendix 3. TRF index (type 5) by sub-national boundary. Appendix 4. Final TRF index values by sub-national boundary. Appendix 5. TRF index in Bangladesh. (ZIP 1995 kb) [file 12879_2016_2074_MOESM1_ESM.zip › A3-1R2.tif]
